# Reservoir assessment, source rock evaluation, and 1D basin modeling in Azraq, Dead Sea, Sirhan, Jafr, and Risha basins, Jordan

**DOI:** 10.1038/s41598-026-52646-w

**Published:** 2026-05-23

**Authors:** Sherif Farouk, Fayez Ahmad, Abdelrahman Qteishat, Souvik Sen, Mohamed Fagelnour, Shu Jiang, Mohamed Arafat, Khaled Al-Kahtany, Mohammad Abdelfattah Sarhan

**Affiliations:** 1 Exploration Department, Egyptian Petroleum Research Institute (EPRI), Nasr City, 1 Ahmed El-Zomor Street, Cairo, 11727 Egypt; 2 Department of Earth and Environmental Sciences, Prince EL-Hassan Bin Talal Faculty for Natural Resources and Environment, The Hashemite University, Zarqa, Jordan; 3 Chemical Engineering Department, Al-Huson University College, Al- Balqa’ Applied University, Irbid, Jordan; 4 Department of Petroleum Engineering, Indian School of Mines), Indian Institute of Technology, Dhanbad, 826004 Jharkhand India; 5 Reservoir Technical Services (RTS), Baker Hughes, Mumbai, Maharashtra India; 6 Khalda Petroleum Company, Cairo, Egypt; 7 School of Sustainable Energy, China University of Geosciences, Wuhan, 430074 China; 8 State Key Laboratory of Petroleum Resources and Engineering, China University of Petroleum (Beijing), Beijing, 102249 China; 9 Geology Department, Faculty of Science, Qena University, Qena, 83523 Egypt; 10 Geology and Geophysics Department, Geological Studies Center, College of Science, King Saud University, Riyadh, Saudi Arabia; 11 Geology Department, Faculty of Science, Damietta University, Damietta, 34517 Egypt

**Keywords:** Petrophysical analysis, Source rock evaluation, 1D basin modeling, Onshore Jordan, Environmental sciences, Solid Earth sciences

## Abstract

This study presents a coherent petrophysical analysis of selected wells in the Azraq, Dead Sea, Sirhan, and Risha basins to define reservoir quality and hydrocarbon prospectivity in diverse geological settings. The application of a consistent set of petrophysical cutoffs enabled the systematic identification and delineation of pay intervals in terms of effective porosity, shale volume, fluid saturation, and net pay thickness, providing a robust basis for comparative assessment and prospect ranking. In total, more than 260 m of combined net pay have been assessed and characterized within the Paleozoic to Eocene interval of the studied wells from four different basins. Based on limited thin sections, quick look petrographic characteristics were evaluated. The assessment of source rocks based on Rock Eval pyrolysis data revealed that the potential source rocks are the Ghareb and Wadi Essir formations in the Azraq Basin, the Mudawwara Formation in the Risha, Jafr, and Sirhan basins, and the Dubeidib and Hiswa formations in the Sirhan Basin. The estimated transformation ratio, along with the timing of maturation relative to trap formation and migration pathways, provided a critical discriminator for determining which source intervals are likely to have contributed to the commercial charge. The integrated geochemical and basin‑modeling results emphasized that successful charge prediction requires coupling kerogen quality with robust maturity and burial histories. This cross-basin comparison study highlights the importance of integrating petrophysical analysis with source rock evaluation in informing exploration plans, identifying sweet spots, and managing reservoirs in geologically complex regions.

## Introduction

Systematic hydrocarbon exploration in Jordan is a relatively recent enterprise that has progressed through episodic phases of reconnaissance, targeted surveying, and intensified national programs. Initial subsurface appraisals were undertaken in the immediate post-World War II era, when the Iraqi Petroleum Company (IPC) completed the first comprehensive reconnaissance of Jordan’s petroleum potential^1,2^. Subsequent activity between 1955 and 1978 primarily consisted of geophysical reconnaissance, principally 2D seismic, gravity, and magnetic surveys conducted by international companies in collaboration with the Natural Resources Authority. Although approximately twenty exploratory wells were drilled during this interval, none yielded commercial accumulations; nevertheless, shows of oil and gas in several wells indicated working petroleum systems and the presence of potential source intervals^1,3^. A decisive shift occurred in the early 1980s when the Jordanian government, motivated by energy security concerns and the oil price shocks of the 1970s, financed a national exploration campaign. This program expanded seismic coverage to some 30,000 km of 2D data and supported the drilling of more than eighty wells across multiple sedimentary basins, culminating in the country’s first commercial discoveries. The Hamzeh oil field (Azraq Basin), discovered in 1984, produces light crude from mid-Cretaceous carbonate reservoirs (Cenomanian–Turonian Hummar and Shueib formations, equivalents of highly productive carbonate units such as the Mishrif and Mauddud in the Arabian Gulf) and represents Jordan’s inaugural oil production, albeit on a modest scale^1,4^. The Risha gas field, discovered in 1987 in northeastern Jordan, constitutes the nation’s most significant hydrocarbon accumulation to date. Risha produces dry gas from deeply buried Ordovician sandstones (Dubeidib Formation), charged from Silurian black shales; early reserve estimates have been revised upward substantially, and production was initiated in 1989, which now supplies a dedicated power facility via a combination of vertical and horizontal wells^4,5^. These two discoveries demonstrate that viable petroleum systems exist in Jordan, but they also underscore the episodic and localized nature of commercial success.

Despite these two discoveries, Jordan’s overall exploration success is low relative to its hydrocarbon-producing neighbors. More than 120 exploration wells have been drilled nationally, yet only these two fields have yielded sustained commercial output. Continued exploration by international operators through the 1980s and 1990s failed to deliver additional commercial discoveries, a shortfall that reflects a combination of complex structural geometries, relatively small and compartmentalized sedimentary basins, and frequently marginal reservoir quality, conditions that particularly challenge exploitation of tight-gas systems. One such example is the Dead Sea Basin. Exploration of the Dead Sea Basin on the Jordanian margin has, to date, yielded limited commercial success, despite abundant surface and subsurface indications of hydrocarbons. Observations range from oil seeps and tar sands to asphalt clasts and accumulations of heavy oil, commonly associated with fault zones and coastal sedimentary deposits. Small gas discoveries (for example, Kidod and Zohar) have been documented in the Israeli sector of the basin, but analogous commercial accumulations have not been realized on the Jordanian side. Previous studies attribute this disparity to challenges in structural targeting and suboptimal well testing and evaluation practices, which have together hindered the effective identification and appraisal of viable traps and charge in the Jordanian portion of the basin^1,4,6^. In response to these constraints, recent exploration emphasis has shifted toward unconventional resources, most notably extensive Maastrichtian oil‑shale deposits concentrated in the Muwaqqar Chalk Marl Formation, where local total organic carbon (TOC) values exceed 20%. Concurrently, attention is increasing on under-explored, frontier petroleum systems that may host significant undiscovered resources. Targets include Cambrian clastic sequences within the Jafr Rift, Triassic–Jurassic successions in northern Jordan, and potential Eocene-Miocene targets in the shallow stratigraphy. Although these plays remain sparsely tested, their structural configurations, source rock potential, and analogies to productive provinces in adjacent countries, such as Syria, Iraq, and Saudi Arabia, suggest they merit a focused geological, geophysical, and geochemical appraisal.

**Fig. 1:**
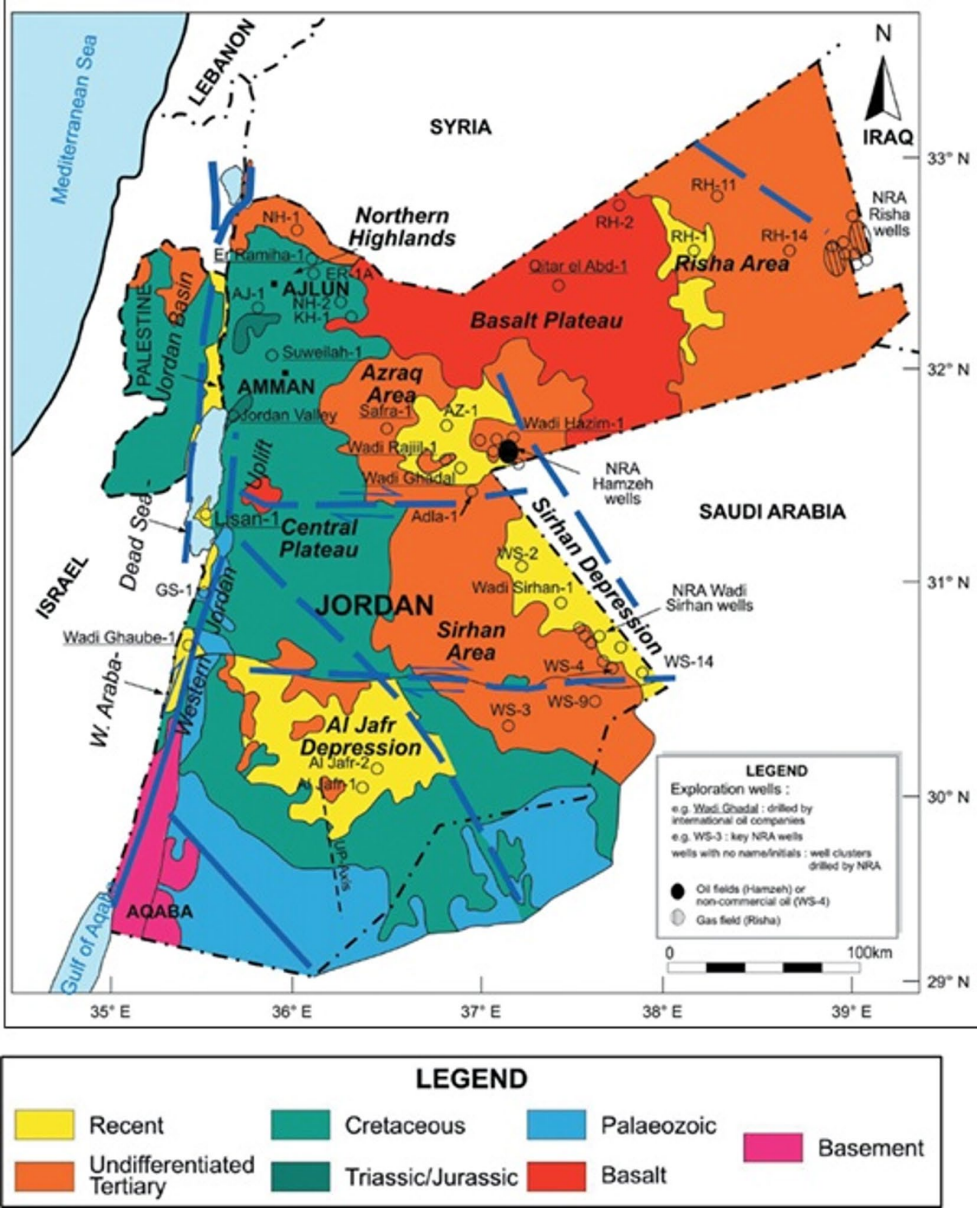
General map shows the locations of the sedimentary basins, drilled wells, and hydrocarbon fields in Jordan, modified after Lüning and Kuss^4^, Beydoun et al.^1^, and Jordanian Natural Resources Authority^2^.

This study integrates wireline logs, petrographic thin sections, and organic geochemical data to refine reservoir and source rock characterization across various petroleum basins of Jordan. This study evaluates reservoir quality and hydrocarbon prospectivity within selected wells from the Azraq, Dead Sea, Sirhan, and Risha basins (Fig. 1). Utilizing the wireline log-based quantitative petrophysical assessments of effective porosity, shale volume, hydrocarbon saturation, and pay thickness, the research seeks to characterize the productive reservoir intervals for potential hydrocarbon development. Complementary geochemical and basin-modeling analyses are incorporated to constrain the source-rock potential and maturation history. Rock-Eval pyrolysis provides kerogen typing and generative potential, while one-dimensional burial-history models, calibrated with vitrinite reflectance where available, establish thermal maturity, timing of hydrocarbon generation, and likely phase evolution. Integrating petrophysical, petrographic, and basin-model outputs, therefore, yields a coherent, risk-informed assessment of both reservoir and charge elements, supporting targeted exploration and optimized development strategies in Jordan’s principal sedimentary basins.

## Geological setting

Jordan’s structural framework is dominated by two principal regional trends, expressed as major fault systems that strike broadly south–north and southeast–northeast. These lineaments originated during the assembly and stabilization of the Arabian Plate and are genetically linked to the development of the Sinai triple junction, from which the Wadi Araba–Dead Sea–Jordan Valley rift, the Najd fault network, and the Sinai structural trend propagated; these Precambrian fabrics have exerted a persistent first‑order control on subsequent deformation and basin architecture^7^. Following the Infra-Cambrian extension associated with the Pan‑African (East African–Antarctic) orogeny, the region experienced a relatively quiescent Paleozoic, punctuated by Late Carboniferous compressional reactivation during the Hercynian event that produced regional uplift, erosion of Upper Paleozoic strata, and reactivation of inherited structural trends^8,9^.

**Fig. 2:**
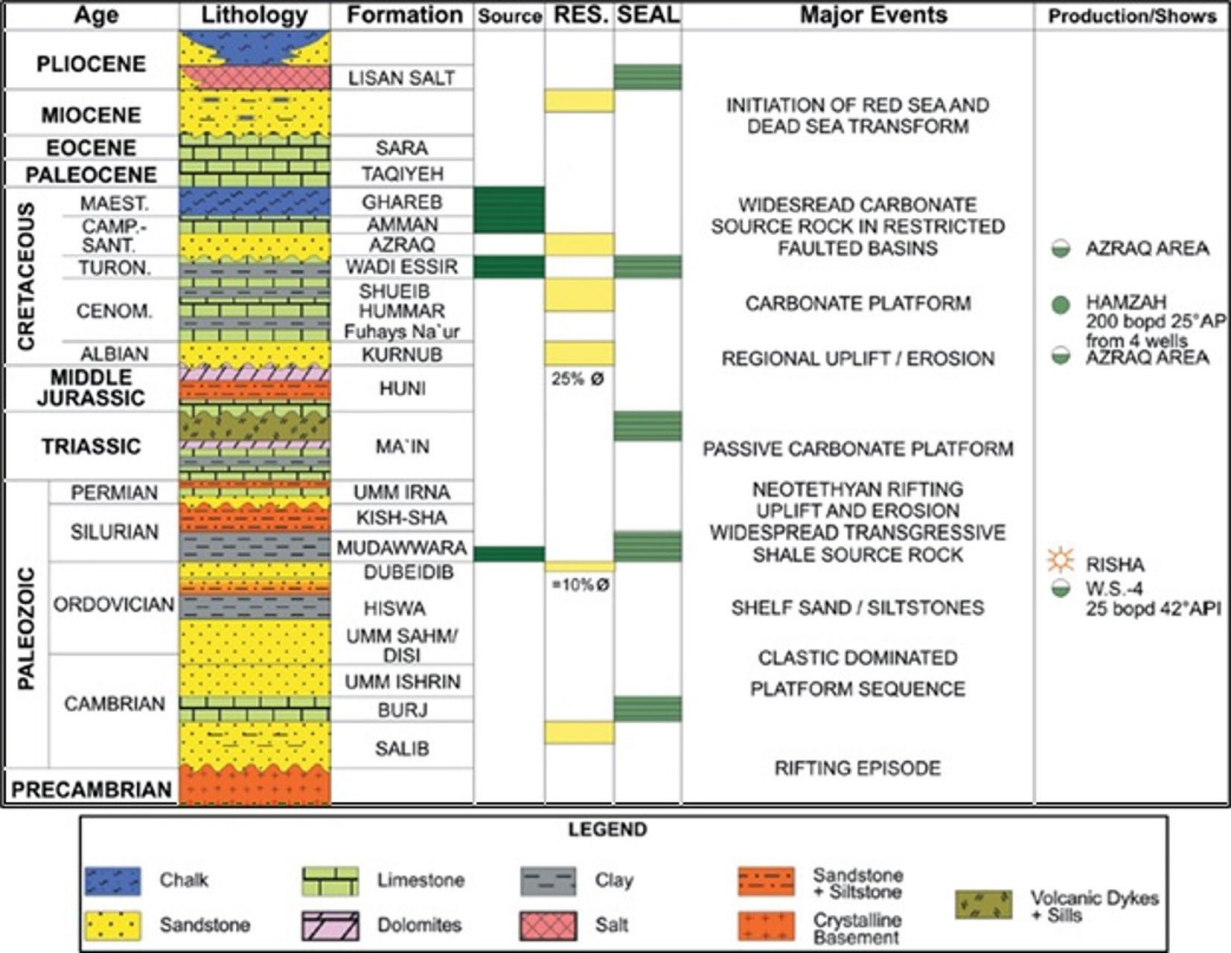
Stratigraphic column of Jordan highlighting key play parameters, major tectonic events, and hydrocarbon occurrences, modified after Lüning and Kuss^4^ and the Jordanian Natural Resources Authority^2^.

Fig. 1 presents the various sedimentary basins of Jordan, along with their associated structural elements. A regional lithostratigraphic chart of onshore Jordan is presented in Fig. 2. The sedimentary fill above the Precambrian basement attains thicknesses of ~6 km. It comprises a Cambrian–Eocene succession deposited unconformably on the crystalline crust^10^. Carbonate facies predominate in the Triassic, Cretaceous, and Paleogene intervals, recording repeated transgressive phases related to Tethyan incursions and the development of an extensive carbonate platform across the region^11^. The boundary is marked by the top of the Silurian Khisheh Formation, which separates the Paleozoic siliciclastic succession from overlying Mesozoic dolomites^10^. The Paleozoic package is dominated by terrestrial to shallow‑marine siliciclastics deposited in repeated transgressive–regressive cycles^12^. The Lower to Middle Cambrian Salib Formation consists predominantly of fluvial to shallow marine sandstones formed in continental facies. The Middle Cambrian Burj Formation records the earliest documented marine transgression in the Jordan area^13^. In contrast, the Upper Cambrian–Ordovician interval is characterized by predominantly non‑marine shale–sand successions that host the principal gas reservoirs of the Risha Field^1,14^. Regional uplift and erosion have removed much of the Upper Paleozoic section within the study area. The Silurian Mudawwara Formation shales are the source rock of the region^10^, deposited during a large-scale transgression in the sea that started due to the melting of Late Ordovician glaciers during Early Silurian times, leading to widespread deposition over Jordan^15^.

The Shueib Formation (Late Cenomanian) of the Middle Cretaceous Ajlun Group is a shallow marine to intrashelf, restricted-basin-dominated carbonate sequence. This unit is of particular interest due to its high levels of total organic carbon (TOC), as observed in outcrop sections near Wadi Karak, where TOC content reaches significant levels with favorable source rock potential^5,16^. Turonian Wadi Sir Formation (commonly known as “Wadi Essir” in previous literature) is a significant component of the upper Ajlun Group. It plays both source and reservoir roles in the petroleum systems of Jordan. This succession is dominated by limestones and marls deposited in a narrow marine setting under suboxic to anoxic conditions. The most notable presence of oil indications within the Wadi Essir Formation is in well WR-1, which demonstrates that the formation can function as a self-sourcing reservoir^1,17^. The Paleocene–Eocene Umm Rijam Formation, consisting of Paleocene to early Eocene marls and chalks, is among the youngest Jordanian sedimentary units with reservoir potential. It is part of the highest sedimentary sequence and typically corresponds to high-quality oil shale intervals, notably in central and northern Jordan^4^.

## Data and methods

### Petrophysical analysis

Wireline logs have been the primary inputs for the quantitative petrophysical analysis of the six wells presented in this study. Two wells were assessed from the Azraq Basin: HZ-15 encountered the Late Cenomanian Shueib reservoir, and Rajil-2 penetrated the Early Turonian Wadi Essir reservoir. The Cambrian Salib and Early Eocene Umm Rijam reservoirs were assessed from the wells AH-2 and Isaal-1, respectively, drilled in the Dead Sea Basin. Well WS-10 is the representative of Sirhan Basin, which was also drilled in the Salib Formation. The most extensive dataset was available from the Risha Field, where well RH-3 intersected producing zones in the Risha, Dubeidib, and Umm Sahm formations. These wells were selected based on the availability and completeness of the data. These wells provide valuable insights into reservoir heterogeneity in structurally and stratigraphically complex basins in Jordan, enabling an excellent appraisal of reservoir quality and hydrocarbon potential.

The suite of wireline logs used in this study includes gamma ray (GR), deep resistivity (LLD), neutron porosity (NPHI), and bulk density (RHOB). These logs were used to estimate significant petrophysical parameters, including water saturation (Sw), effective porosity (PHIE), and shale volume (VSH). The gamma ray log was specifically employed to approximate Vsh^18^:

1 $$Vsh=\frac{GR-GRmin}{GRmax-GRmin}$$

GRmin and GRmax represent the minimum and maximum gamma ray values, respectively, recorded within the targeted formation interval. Effective porosity (PHIE) was calculated by integrating the estimated shale volume (Vsh), neutron porosity (NPHI), and total porosity derived from density logs. Water saturation (Sw) was determined using the Indonesian model^19^:

2 $$Sw= {\left\{\frac{\sqrt{\frac{1}{Res}} }{\left\{\left(\frac{Vs{h}^{1-0.5 Vsh}}{\sqrt{Rsh}}\right)+\sqrt{\frac{\Phi {e}^{m}}{a Rw}}\right\}}\right\}}^\frac{2}{n}$$

For SW estimation, we have considered a shale resistivity (Rsh) of 5 Ωm, connate water resistivity (Rw) of 0.03 Ωm, a tortuosity factor (a) value of 1, and cementation and saturation exponents (m and n) both assumed to be 2. To delineate the pay zones, the following petrophysical cut-off values were applied: water saturation ≤ 60%, hydrocarbon saturation ≥ 40%, effective porosity ≥ 7%, shale volume ≤ 30%, and net pay thickness ≥ 4 meters.

### Petrographical analysis

The assessment of the reservoir by means of thin sections under plane- and crossed-polarized light provides critical information about the mineral components, texture, and both visual porosity and permeability. Cores available from the Cambrian Salib reservoir in well WS-3 (Sirhan Basin), Lower Turonian Wadi Essir Formation from Rajil-2, Shueib Formation in the HZ-15 (Azraq Basin), and Dubeidib Formation in the RH-3 (Risha Basin) were utilized to prepare petrographic thin sections. An Olympus BX53P petrographic polarizing microscope was used to study the thin sections. Blue-dyed epoxy resin was used to highlight porosity in the thin sections^20^; no additional stains were applied. Limited scanning electron microscopy (SEM) images were available from the Shueib Formation (HZ-15) and Dubeidib Formation (RH-3). The Quanta FFG-450 SEM instrument was used for SEM analysis.

### Geochemical analysis

The analyses of Total Organic Carbon (TOC), Rock-Eval pyrolysis, and vitrinite reflectance (Ro%) provide an approach for evaluating the organic matter content, thermal maturity, and hydrocarbon generation potential of the source rock intervals^21,22^. These analyses were performed in 2007 by Petrel Resources Company and NPC in Jordan. For TOC analysis, samples were pulverized into a fine powder (less than 200 mesh) and analyzed using a LECO C230 carbon analyzer. The source rock quality was evaluated based on the TOC (wt.%) cut-off values^23^. The samples with high TOC values (higher than 0.5%) were selected for further Rock-Eval pyrolysis using a Rock-Eval 6, which measured S1, S2, S3, and Tmax^21,22^. The Kerogen type and character of expelled products is investigated using the hydrogen index (HI) where Kerogen type-I has HI value > 600 mg HC/g TOC, Kerogen type-II has HI value from 300 to 600 mg HC/g TOC, Kerogen type-II/III has HI from 200 to 300 mg HC/g TOC, Kerogen type-III has HI value from 50 to 200 mg HC/g TOC, and Kerogen type-IV has HI value from <50 mg HC/g TOC^24^. The modified van Krevelen diagram, where HI is plotted against OI, is used to depict the Kerogen type^25^. The TOC-HI plot was used to infer the type of hydrocarbons generated^26^.

The thermal maturity of the samples was assessed through vitrinite reflectance (Ro%), which was available for a total of 25 samples across the four studied wells. Vitrinite reflectance and microscopic kerogen analyses were conducted on <10 μm sieved kerogen particles, mounted in resin blocks and polished with carborundum and alumina, using a calibrated Leica Orthoplan microscope photometer with a variable measuring diaphragm (MPV) under oil immersion, with measurements taken on chitinozoans in the Paleozoic formations^27^. The Ro values were converted into vitrinite Ro-equivalent using the equation [VitRo-eq=(ChRo-0.08)/1.05]^28^. The crucial phase of petroleum generation is defined by the transformation ratio, TR = 50%^29^. TR was modeled in three wells: HZ-4 from the Azraq Basin, RH-23 from the Risha Basin, and WS-3 from the Sirhan Basin.

### 1D basin modeling

A subsidence curve/profile at any drilled well location within a sedimentary basin is a function of multiple corrections of compaction, bathymetry, erosion, long-term fall and rise of sea level, and sedimentary load above the sea level^30^. It is an effective illustration to distinguish the parameters of time, depth, and thermal maturity for the entire sedimentary sequence encountered in the drilled wells. Burial-history models were constructed using the Easy%Ro method^31^. Based on data availability, four wells were selected: HZ-4 in the Azraq Basin, RH-23 in the Risha Basin, WS-3 in the Sirhan Basin, and JF-1 in the Jafr Basin. The models incorporated well tops and thickness data from logs, lithology, thermal maturity, absolute ages for each formation, and unconformities. The 1D basin modeling technique simulates geological events and evolution over time, providing insights into the sedimentary basin’s history and development^25,32–34^.

## Results

### Petrophysical assessment

Table 1 summarizes the petrophysical assessment results of the various reservoirs from the Azraq, Dead Sea, Sirhan, and Risha fields, revealing a broad spectrum of reservoir quality variation across the region.

**Fig. 3:**
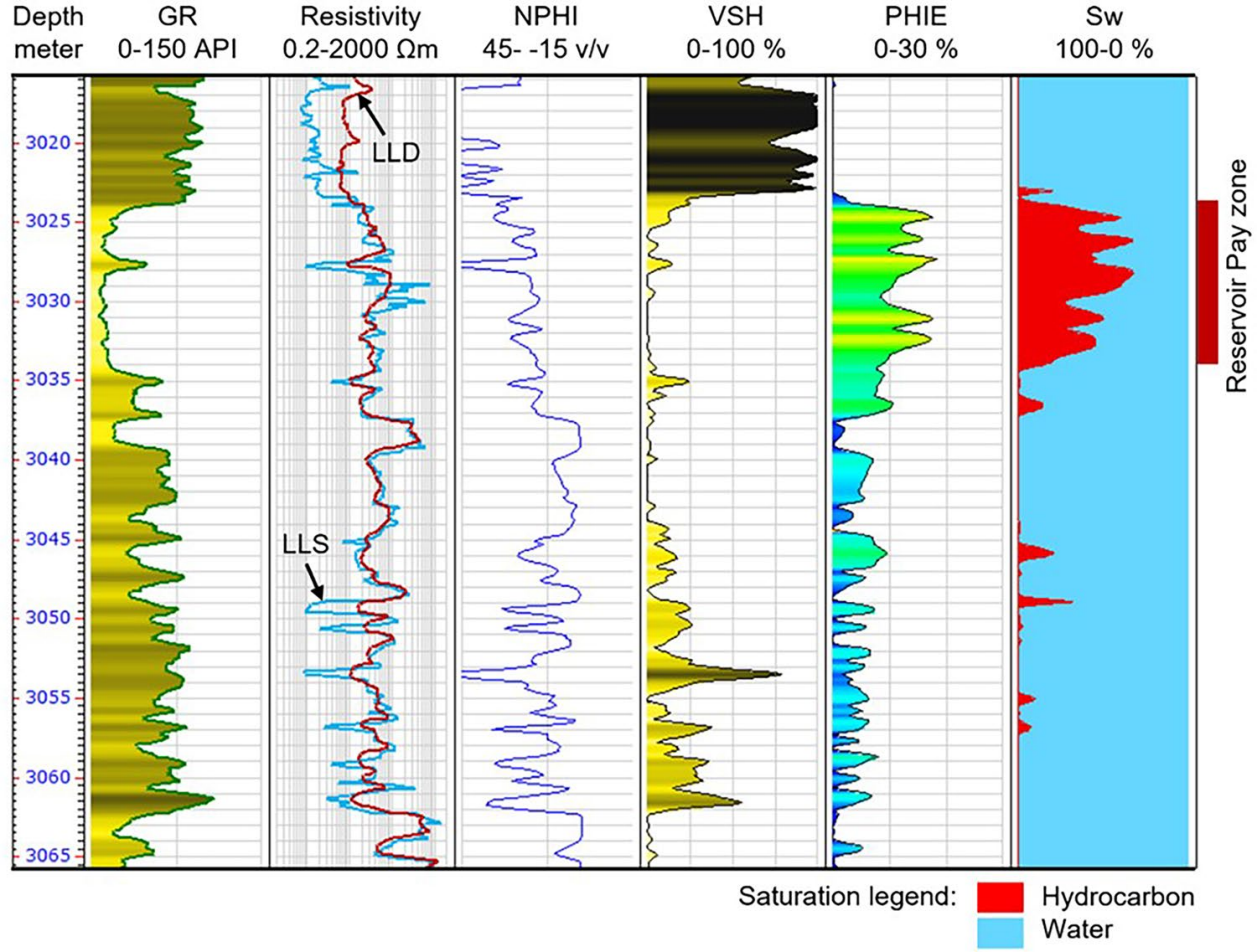
Wireline log signatures and quantitative petrophysical assessment of the upper cenomanian shueib formation in the HZ-15 Well, Azraq Basin. The red bar delineates the dolomitic reservoir pay zone. VSH = shale volume, PHIE = effective porosity, and Sw = water saturation.

**Fig. 4:**
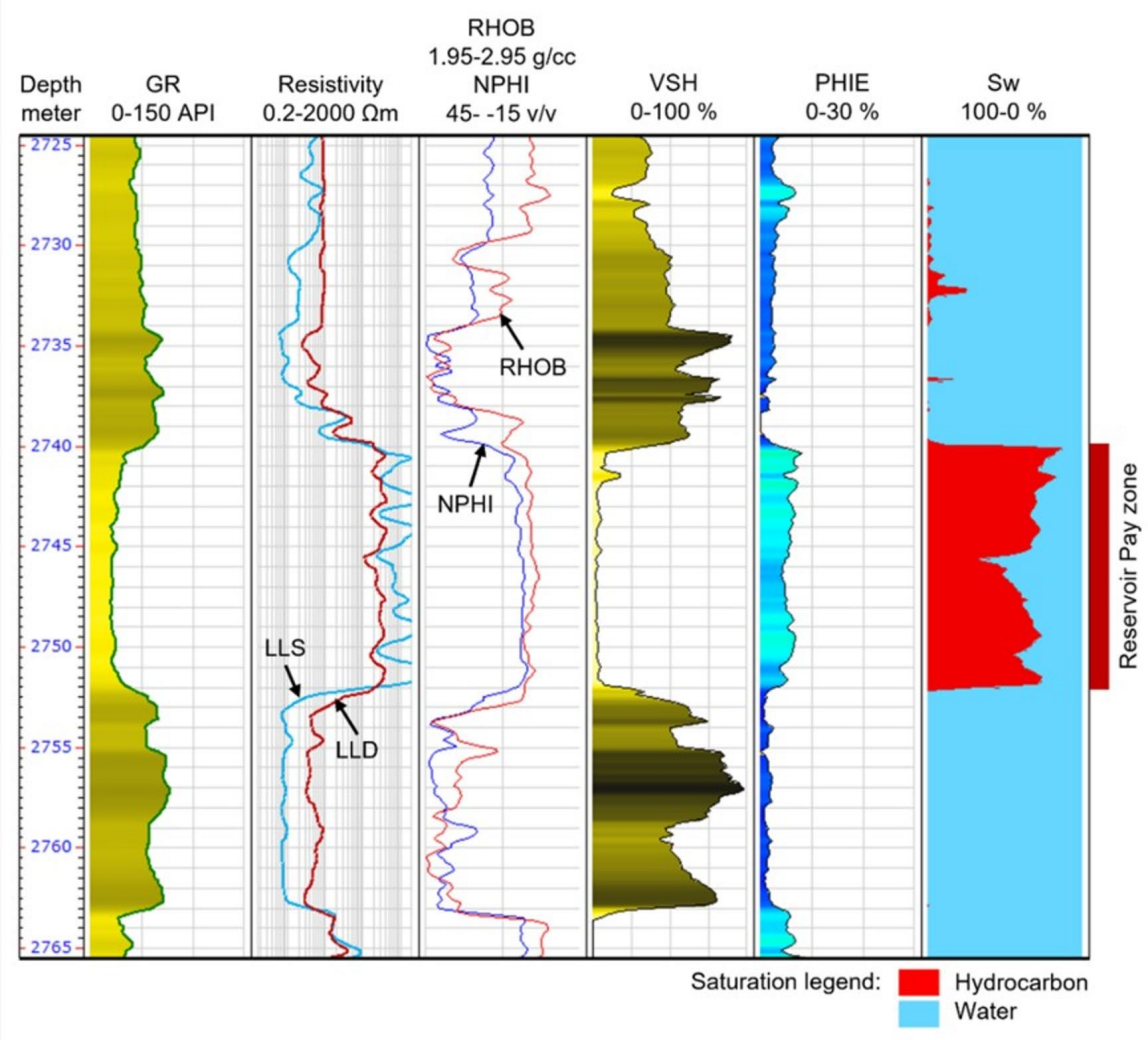
Wireline log signatures and quantitative petrophysical assessment of the Lower Turonian Wadi Essir Formation in the Rajil-2 well, Azraq Basin. The red bar delineates the reservoir pay zone.

**Fig. 5:**
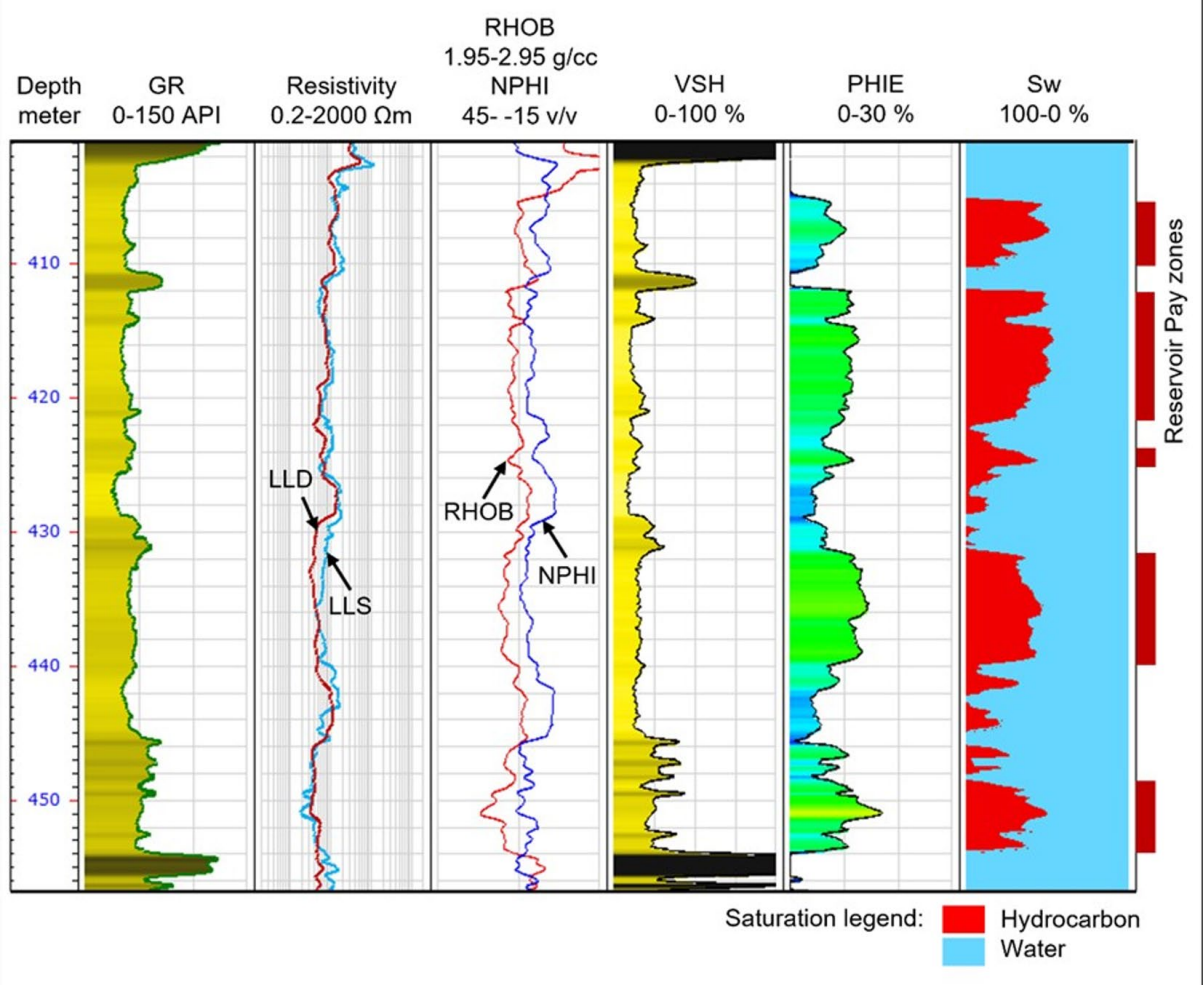
Wireline log signatures and quantitative petrophysical assessment of the cambrian salib formation in the AH-2 well, Dead Sea Basin. The red bars delineate the sandstone reservoir pay zones.

**Fig. 6:**
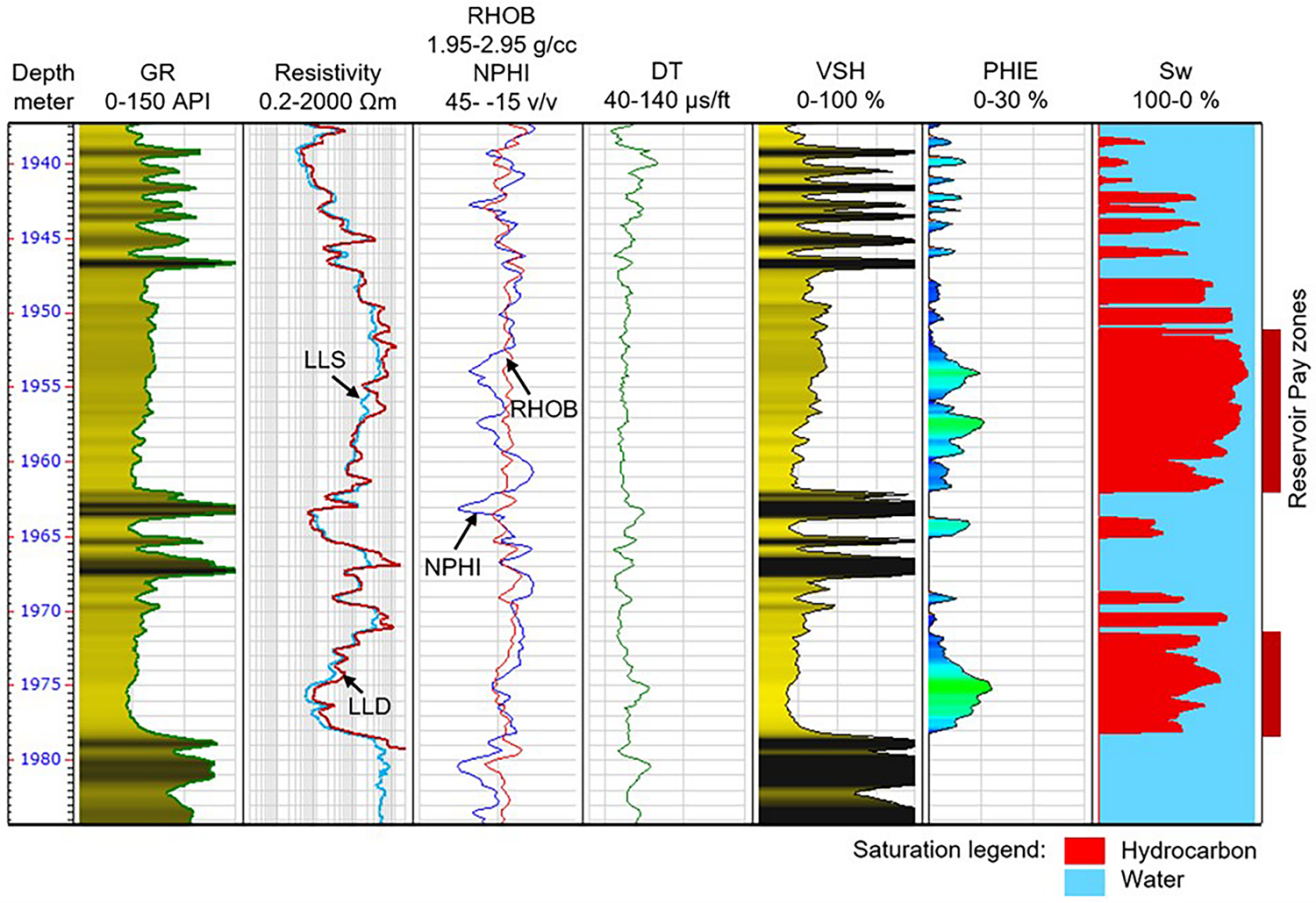
Wireline log signatures and quantitative petrophysical assessment of the lower eocene umm rijam formation in the Isaal-1 Well, Dead Sea Basin. The red bars delineate the reservoir pay zones.

**Fig. 7:**
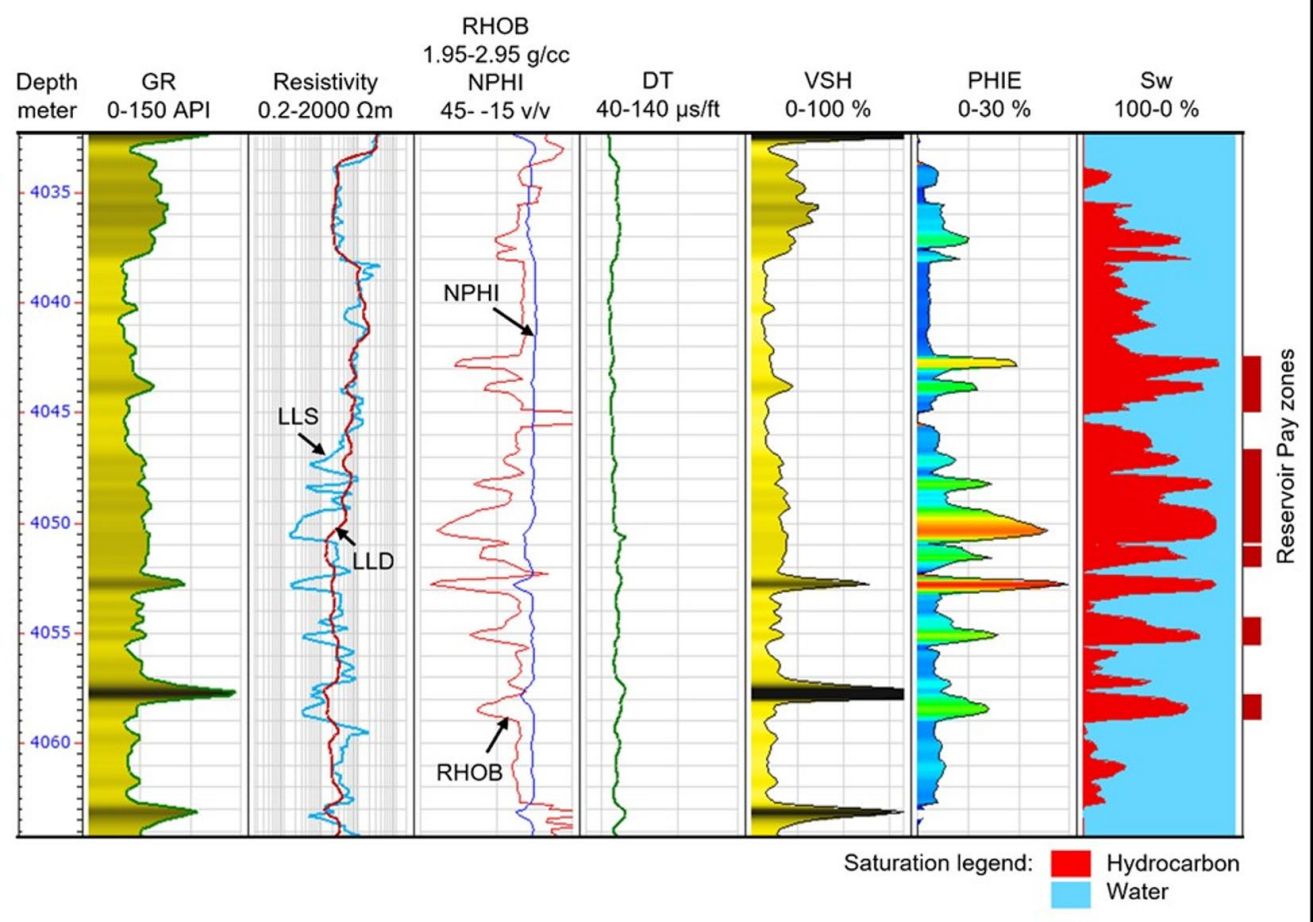
Wireline log signatures and quantitative petrophysical assessment of the cambrian salib formation in the WS-10 well, Sirhan Basin. The red bars delineate the sandstone reservoir pay zones.

**Fig. 8:**
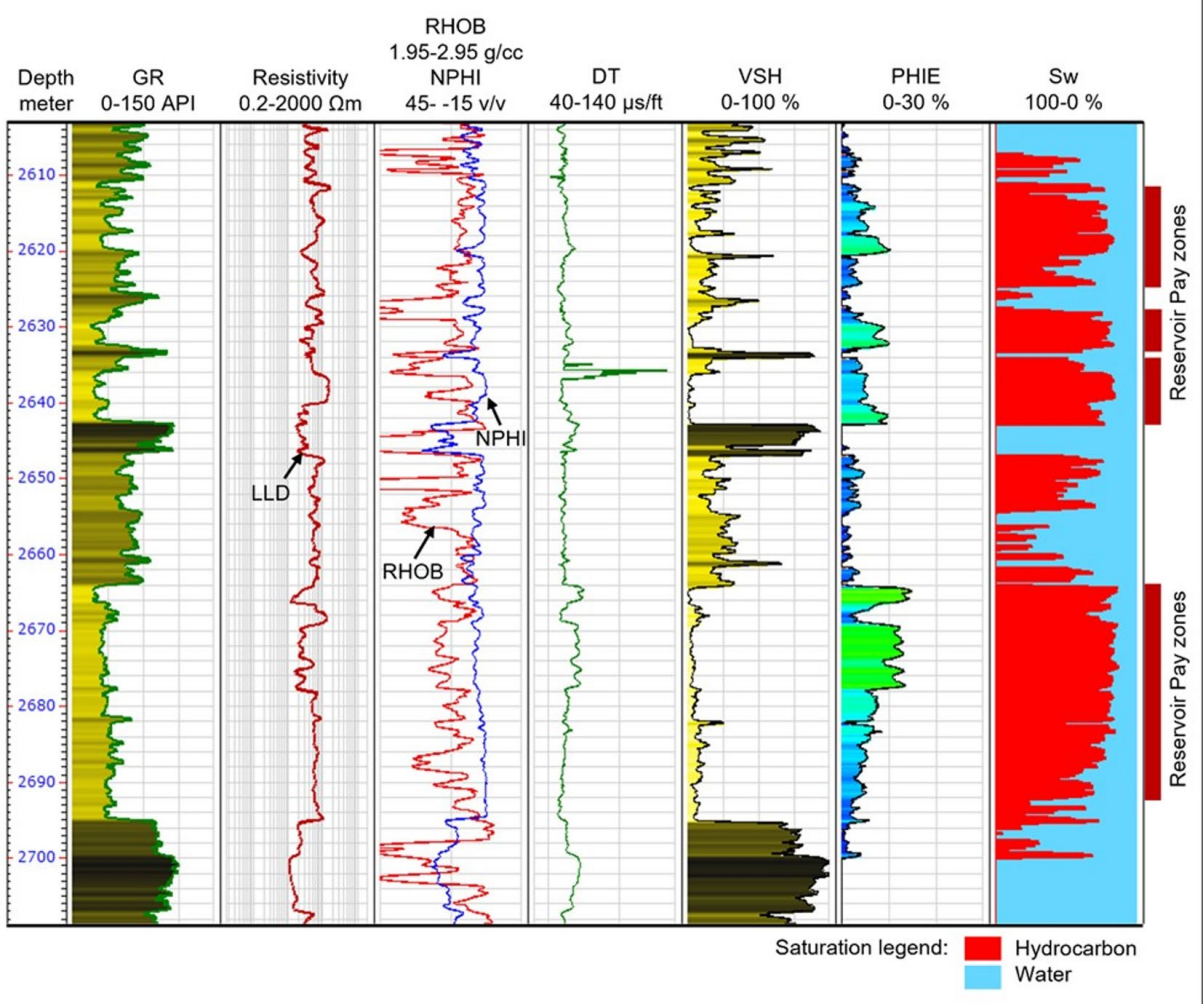
Wireline log signatures and quantitative petrophysical assessment of the ordovician risha formation in the RH-3 well, Risha Field. The red bars delineate the sandstone reservoir pay zones, modified after Farouk et al.^7,36^.

**Fig. 9:**
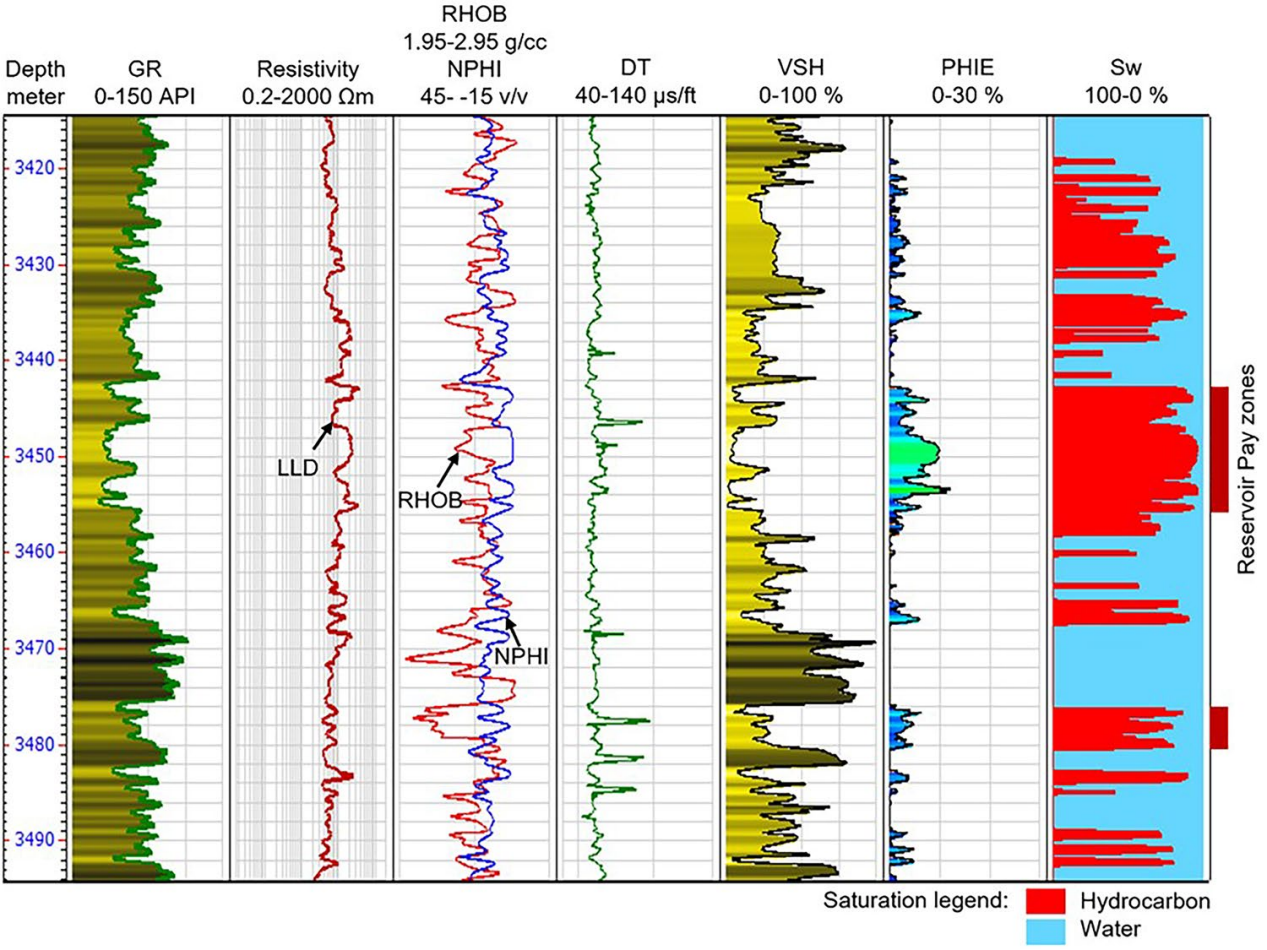
Wireline log signatures and quantitative petrophysical assessment of the ordovician dubeidib formation in the RH-3 well, Risha Field. The red bars delineate the sandstone reservoir pay zones, modified after Farouk et al.^7,36^.

**Fig. 10:**
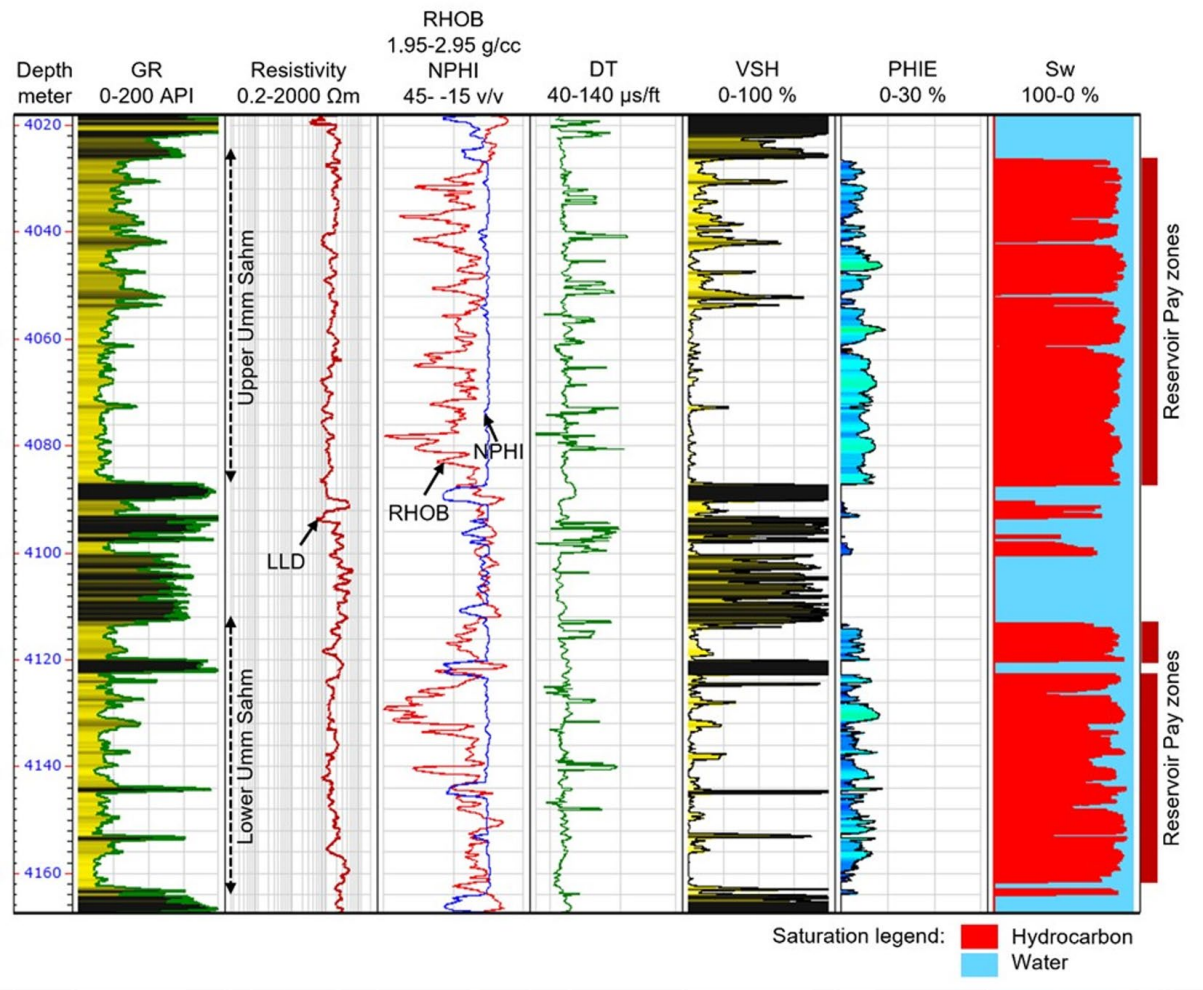
Wireline log signatures and quantitative petrophysical assessment of the ordovician umm sahm formation in the RH-3 well, Risha Field. The red bars delineate the sandstone reservoir pay zones, modified after Farouk et al.^7,36^.

**Fig. 11:**
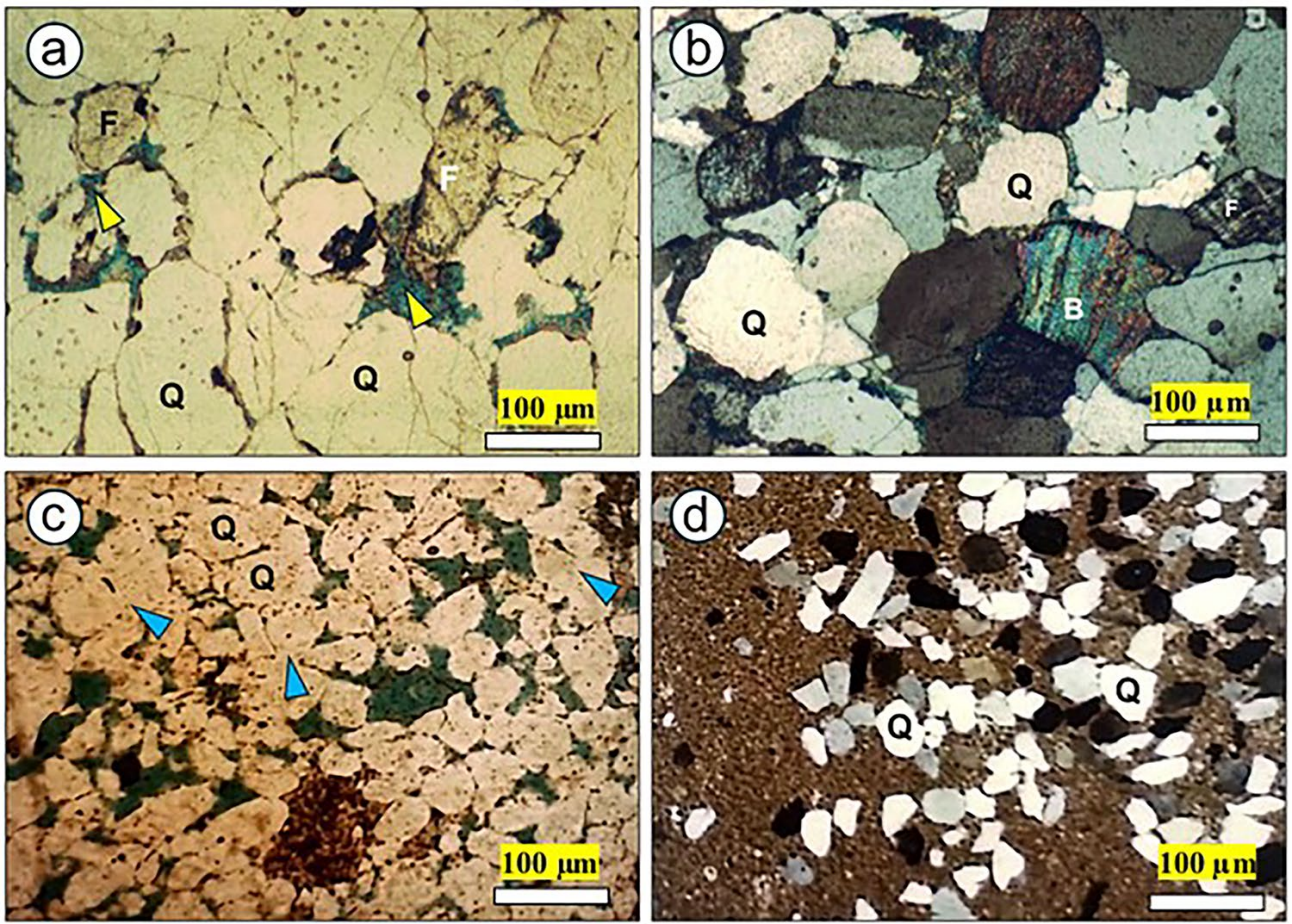
Thin section photomicrographs of reservoir rock samples, Cambrian Salib sandstone from WS-3, depth 3715 m: (**a**) under plane-polarized light, width of view 3.2 mm, consists of quartz (Q), feldspar (F), secondary porosity due feldspar dissolution (marked by yellow arrow), (**b**) under crossed-polarized light, width of view 2.4 mm, coarse, poorly sorted arkose with feldspar, biotite mica (B), and clays; Lower Turonian Wadi Essir Formation from Rajil-2 Well, depth 2745 m: under (**c**) plane-polarized light and (**d**) crossed-polarized light, indicating medium-grained sandstone with good to moderate visible porosity, quartz overgrowth cementation (marked by blue arrow) diminishing primary porosity, however dissolution of detrital grains has increased porosity. The detrital components include silty mudstone interclasts.

- Azraq Basin: A 10 m reservoir pay zone is inferred within the Late Cenomanian Shueib Formation in the well HZ-15 (Fig. 3). The zone exhibits good petrophysical properties, characterized by low VSH (< 5%), 15% effective porosity, and ~50% Sw (Fig. 3). These parameters indicate a clean reservoir sandstone with a high likelihood of hydrocarbon productivity. Another studied well Rajil-2 penetrated a 12 m thick pay zone within the Early Turonian Wadi Essir Formation (Fig. 4). This interval shows a relatively lower PHIE (< 10 %); however, based on the good cleanliness (VSH < 5 %) and low Sw (~ 40 %) (Fig. 4), the interval translates to a fair reservoir with low water cut possibilities.
- Dead Sea Basin: The well AH-2 encountered a cumulative of ~31 m of reservoir pay thickness within the Cambrian Salib Formation, distributed across three zones, characterized by 10-15 % PHIE, varying VSH (6-25 %), and SW ≤ 60 % (Fig. 5). Out of these three zones, upper two intervals have superior petrophysical properties and cleaner than the bottom interval. The second well from the Dead Sea Basin, Isaal-1, drilled through the Umm Rijam Formation and intersected two pay intervals (Fig. 6). Both intervals exhibit poor porosity (≤ 10 %) and VSH varying between 20-30 %, but host high hydrocarbon saturation (Sw 20-45 %) (Fig. 6).
- Sirhan Basin: The studied well WS-10 encountered around 10 m of net pay within the Cambrian Salib Formation at a depth around 4040-4050 m (Fig. 7). The zone had moderate reservoir characteristics with 10 % PHIE, 15 % VSH, and Sw around 50 %. These parameters suggest an average to good storage capacity reservoir and hydrocarbon mobility.
- Risha Basin: The most prospective and promising hydrocarbon-bearing intervals were encountered in the Risha field. He studied well RH-3, which drilled multiple pay zones in the Paleozoic interval. Figs. 8-10 present the petrophysical assessment of potential sandstone reservoir pay zones within the Ordovician Risha, Dubeidib, and Umm Sahm formations (Figs. 8-11). The Risha Formation shows two promising zones. The 32 m thick upper zone between 2610-2642 m has PHIE 8-10 %, VSH 10-20% and Sw~ 20-30 % (Fig. 8). The lower Risha reservoir between 2665-2692 m exhibits superior petrophysical qualities with 12 % PHIE, 5 % VSH and 25 % Sw (Fig. 8). Two potential pay zones are identified within Dubeidib, which are relatively tight, PHIE 7-10 % dominantly and VSH 10-25 % (Fig. 9). Both the zones exhibit high hydrocarbon saturation (Sw around 20-40 %) (Fig. 9). The Umm Sahm offers two massive pay zones within amalgamated sands, with a cumulative net pay of 108 m (Fig. 10). A 25 m thick shale separates these two intervals. The potential Upper Umm Sahm pay is 63 m thick, while the lower pay zone is 45 m thick. Both intervals are clean (VSH < 10%) with PHIE varying between 8% and 10% (Fig. 10) and exhibit very high hydrocarbon saturation (Sw ~ 20%). The highly saturated reservoirs indicate that the Risha Field has excellent potential for hydrocarbons.

**Table 1:** Summary of the average calculated petrophysical parameters for the reservoir intervals in the analyzed wells. The applied cut-off values to delineate the pay zones are water saturation ≤ 60%, hydrocarbon saturation ≥ 40%, effective porosity ≥ 7%, shale volume ≤ 30%, and net pay thickness ≥ 4 meters.

**Area**	**Well** **name**	**Formation**		**Pay intervals**		**Pay thickness (m)**	**Effective porosity %**	**Shale volume %**	**Water saturation %**	**Hydrocarbon saturation %**	**Figure no.**
				From **(m)**	To (m)						
Azraq Basin	HZ-15	Shueib		3025	3035	10	15	3	50	50	3
	Rajil-2	Wadi Essir		2740	2752	12	8	2	40	60	4
Dead Sea Basin	AH-2	Salib		405	422	17	10	6	55	45	5
				432	440	8	13	17	60	40	
				448	454	6	15	25	60	40	
	Isaal-1	Umm Rijam		1950	1960	10	7	30	20	80	6
				1972	1978	6	10	20	45	55	
Sirhan Basin	WS-10	Salib		4040	4050	10	10	15	50	50	7
Risha Field	RH-3	Risha		2610	2642	32	8	10	30	70	8
				2665	2692	27	12	5	25	75	
		Dubeidib		3442	3456	14	10	10	20	80	9
				3476	3480	4	7	25	40	60	
		Umm Sahm	Upper	4025	4088	63	10	5	20	80	10
			Lower	4115	4160	45	8	5	20	80

### Petrographic investigation: reservoir facies analysis

**Fig. 12:**
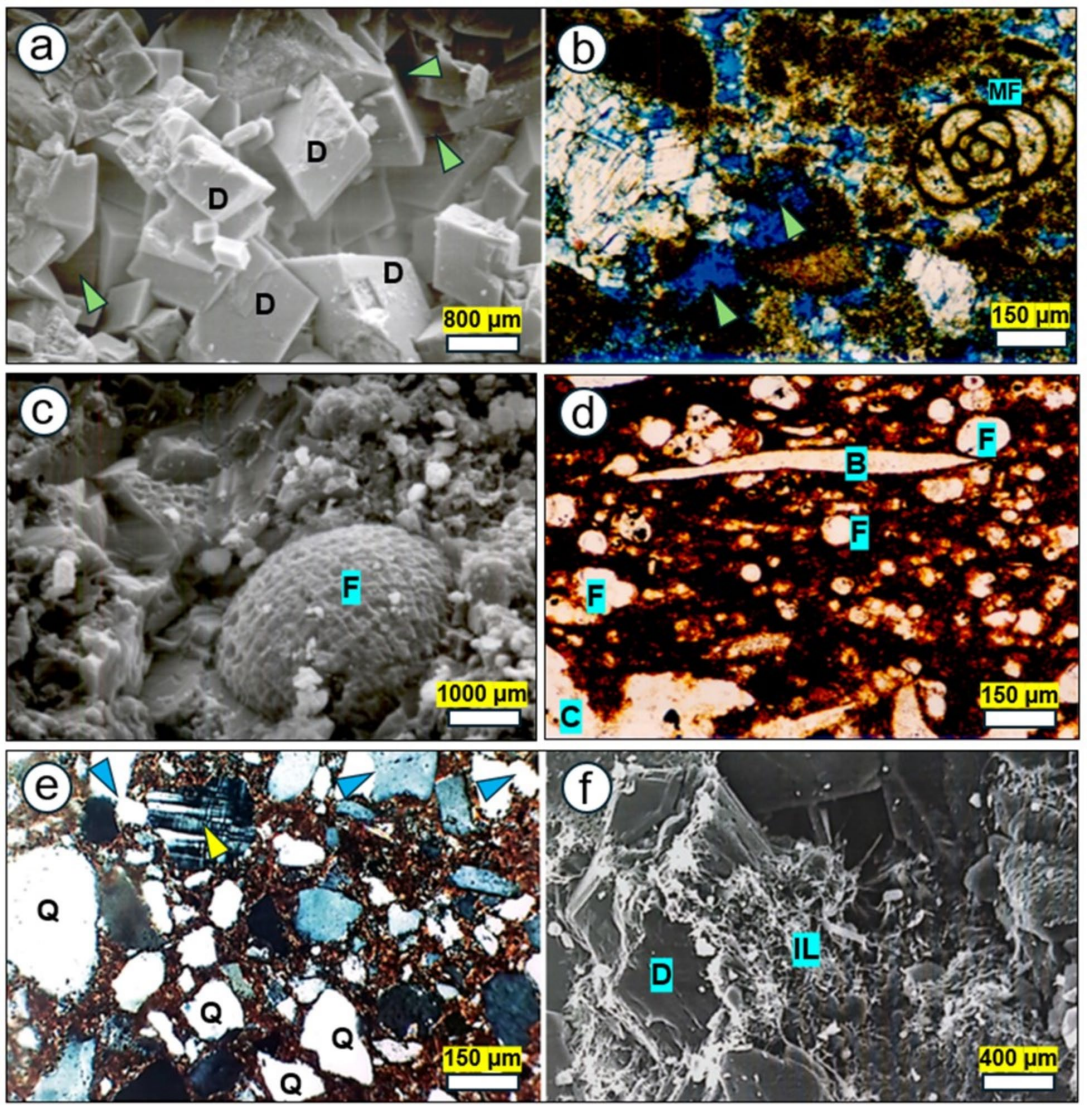
Thin section and SEM photomicrographs of reservoir rock samples, Shueib Formation in the HZ-15 exhibiting (**a**) dolomite rhombs (D) and excellent intercystalline porosity (green arrows) in SEM, (**b**) dolomitized intraclasts and a miliolid Foraminifera (MF) represent the dominant framework grains under plane-polarized light, (**c**) foraminiferal tests (F) are preserved within a larger mass of calcite cement in SEM; (**d**) Wadi Essir Formation in the Rajil-2 (depth 2725 m) showing wackestone with densely packed foraminiferal tests (F), bioclasts (B) and recrystallized calcite (C); Dubeidib Formation in the RH-3 showing (**e**) subfeldspathic arenite with quartz (Q) with authigenic overgrowth (blue arrow), feldspar dissolution (yellow arrow), and (**f**) illite (IL) filaments lining pore throats and coating dolomites (D) in SEM.

The petrographical study of sandstone reservoir thin sections of the Salib Formation in WS-3 well revealed that grains are moderately to well-rounded feldspar and well-rounded to subangular quartz grains, with moderate to high sphericity (Fig. 11a-b). Lithic grains include siltstone and quartzite, while occasional detrital micas include biotite. Matrix includes severe quartz overgrowths and kaolinitic clay cements, which reduce porosity. Visual porosity accounts for 7.6%, which is mostly primary intergranular porosity, and part of it is secondary, resulting from feldspar dissolution (Fig. 11a). The depositional environment is most likely to be fluviatile^35^. The quartz greywacke thin sections of the Wadi Essir Formation in the Rajil-2 well showed medium-grained sandstone with good visible primary intergranular porosity (up to 9 %). Overgrowths and the presence of silty mudstone interclasts reduce primary porosity. The dissolution of detrital grains has contributed to the secondary porosity (Fig. 11c-d). Fig. 12d presents a thin section of Wadi Essir wackestone from Rajil-2 that exhibits densely packed foraminiferal tests and bioclasts. The spherical tests are infilled by blocky calcite cement. Recrystallized calcite and pyrite are present as post-depositional diagenetic minerals (Fig. 12d). The presence of shallow-water foraminifera, coarse bioclastic debris, and decayed algae indicates a shallow marine, low to medium-energy inner shelf environment of deposition (Folk 1951).

The thin section and SEM of shallow marine Shueib carbonates from HZ-15 are presented in Fig. 12a-c, which indicates porous dolomites with excellent intercystalline porosity. Dolomite rhombs range from very finely crystalline to finely crystalline (Fig. 12a). Penetration molds are visible on several rhombs. Although some dolomite rhombs appear interlocked, communication throughout the pore network appears excellent. Thin section indicates dolomitized intraclasts and a miliolid Foraminifera represent the dominant framework grains (Fig. 12b), while SEM indicates micrite-rich matrix surrounding the densely packed foraminiferal tests and bioclasts (Fig. 12c).

Dubeidib thin section and SEM from RH-3 indicate subfeldspathic quartz arenite, with quartz and feldspar as primary framework grains with minor dolomites (Fig. 12e-f). The majority of grains display a tight interlocking mosaic of clasts and overgrowths with minimal porosity development. Although feldspar dissolution contributed to some secondary porosity developments, the SEM image indicates illite filaments lining pore throats and coating dolomites (Fig. 12f). These clay coatings and authigenic quartz overgrowths were inferred as the key diagenetic elements reducing reservoir porosity. The tight nature of Dubeidib reservoir intervals is also depicted in the quantitative petrophysical assessment (Fig. 9). Farouk et al.^7,36^ studied the core samples from the Risha, Dubeidib, and Umm Sahm reservoirs in RH-3 and RH-4 wells. The authors reported that these three reservoirs are fine-grained sandstones, moderately sorted with high mineralogical maturity. The Risha and Dubeidib reservoirs are subarkose, while the Umm Sahm reservoir is composed of quartz arenite. These reservoirs are micro- and mesoporous, with permeability < 1 mD and dominantly < 6% porosity^7^. Thin sections and SEM assessment of the Dubeidib reservoir in RH-4 well revealed that the reservoir is composed of sub-feldspathic arenites, comprising monoclinic quartz, feldspars, and polycrystalline quartz, with minor amounts of detrital clay in the pores and quartz overgrowths that reduce the pore volume^36^.

### Source rock evaluation

**Fig. 13:**
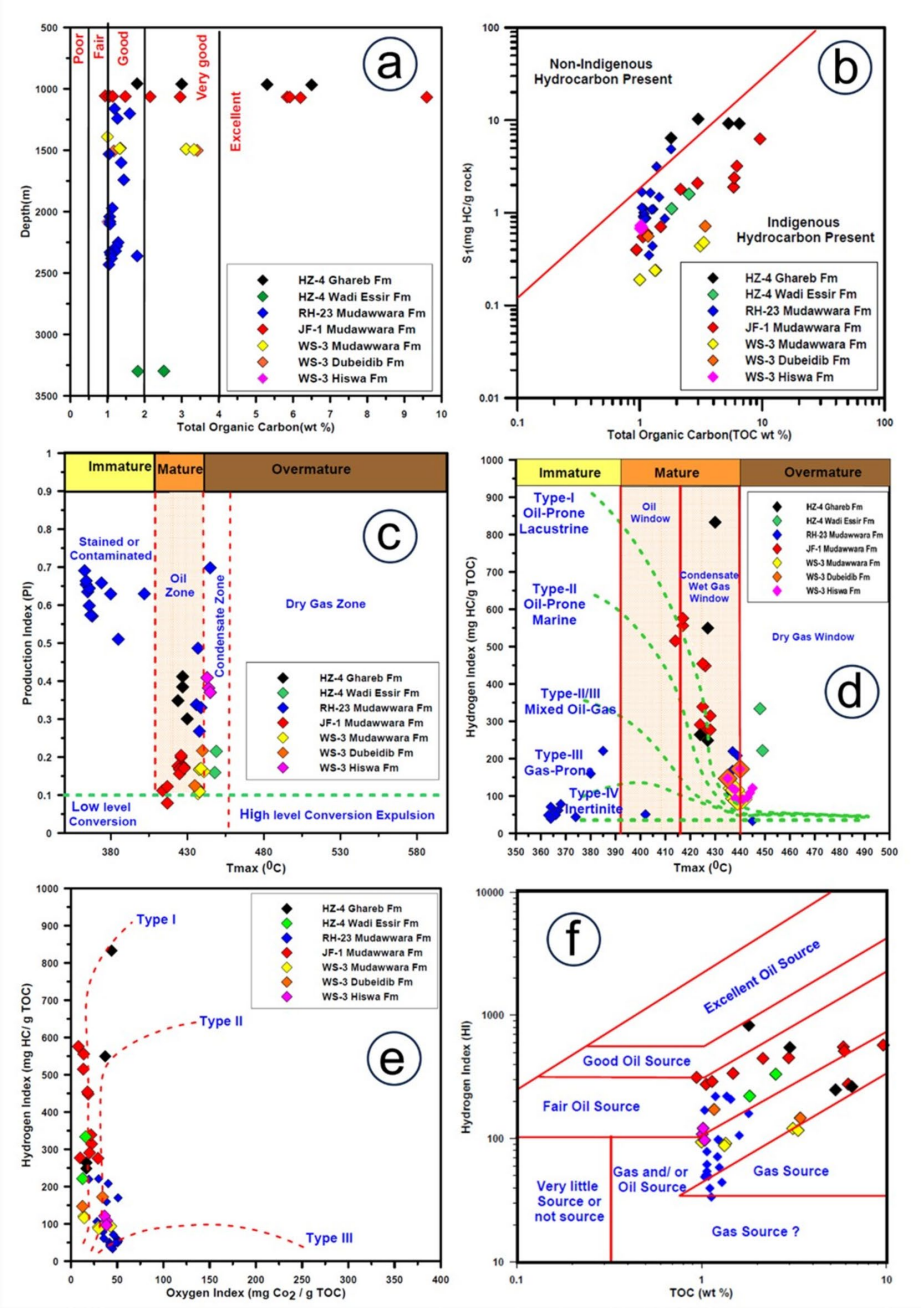
Organic geochemical analysis and source rock assessment: (**a**) TOC vs. Depth diagram (modified after Peters and Cassa, 1994), (**b**) TOC vs. S1 cross plot (modified after Jarvie et al., 2001), (**c**) Tmax vs. production index (PI) cross plot (modified after Delgado et al., 2018), (**d**) Tmax vs. Hydrogen Index (HI) cross plot (modified after Khatibi et al., 2018), (**e**) modified Van-Krevelen diagram (HI vs. OI) (modified after Waples, 1985), and (f) TOC vs. Hydrogen Index (HI) cross plot (modified after Delvaux et al., 1990).

The organic geochemical analysis performed on the drill cutting samples recovered from four wells in four different basins is presented in Table 2. The measured TOC values of Ghareb limestone samples in HZ-4 well in Azraq Basin range from 7.34 to 14.06 wt.%, indicating good to excellent source rock quality (Fig. 13a). The Wadi Essir marl samples in HZ-4 well have TOC~ 1.82-2.52 wt.%, indicating good to very good source rock quality (Fig. 13a). Mudawwara clay samples in RH-23 in Risha Basin range from 1.04 to 1.8 wt.%, exhibiting good source rock quality (Fig. 13a). The same formation in JF-1 of Jafr Basin shows 0.94-9.6 wt.% TOC, which indicates that Mudawwara clay samples have good to excellent source rock qualities (Fig. 12a). Mudawwara Formation is also evaluated in Sirhan Basin, where the clay samples in WS-3 well have TOC values that range from 1 to 3.33 wt. % (good to very good source rock quality). The Dubeidib Formation in WS-3 of Sirhan Basin has TOC values ranging from 1.17 to 3.42 wt. % (good to very good quality), while the Hiswa Formation in the same well has TOC between 1.01 and 1.04 wt. % (good source rock quality) (Fig. 13a).

**Table 2:** Total Organic Carbon (TOC) and Rock-Eval pyrolysis data for Mudawwara Formation in RH-23, Jafr-1, HZ-4 and WS-3 wells. TOC is expressed as a percentage. S1, S2, and S3 represent hydrocarbon yields in mg HC/g rock. Hydrogen Index (HI) is reported in mg HC/g TOC, and Oxygen Index (OI) in mg CO_2_/g TOC. Maximum temperature (Tmax) is in °C. Depths are given as measured depth (MD).

**Well**	**Formation**	**Depth**	**TOC**	**S1**	**S2**	**S3**	**Tmax**	**OI**	**HI**	**PI**	**S2/S3**
		m	wt. %	mg HC/g rock	mg HC/g rock	mg HC/g rock	°C	mg CO2/g TOC	mg HC/g TOC	**-**	**-**
**RH-23 (Risha Field)**	Mudawwara	1160	1.19	0.35	2.62	0.23	437	19.33	220.17	0.12	11.39
		1200	1.6	0.87	1.7	0.45	436	28.13	106.25	0.34	3.78
		1240	1.27	0.44	1.2	0.39	438	30.71	94.49	0.27	3.08
		1530	1.04	1.68	1.77	0.53	437	50.96	170.19	0.49	3.34
		1600	1.37	3.16	3.03	0.41	385	29.93	221.17	0.51	7.39
		1740	1.44	1.48	3	0.58	439	40.28	208.33	0.33	5.17
		1970	1.13	0.88	0.38	0.51	445	45.13	33.63	0.7	0.75
		2040	1.06	0.92	0.54	0.44	402	41.51	50.94	0.63	1.23
		2080	1.07	1.12	0.84	0.37	368	34.58	78.5	0.57	2.27
		2100	1.07	0.89	0.66	0.38	367	35.51	61.68	0.57	1.74
		2250	1.29	1.1	0.57	0.54	374	41.86	44.19	0.66	1.06
		2280	1.25	1.09	0.73	0.63	366	50.4	58.4	0.6	1.16
		2320	1.22	1.65	0.87	0.56	364	45.9	71.31	0.65	1.55
		2330	1.07	1.01	0.58	0.55	365	51.4	54.21	0.64	1.05
		2350	1.09	0.98	0.54	0.55	366	50.46	49.54	0.64	0.98
		2360	1.8	4.9	2.88	0.69	380	38.33	160	0.63	4.17
		2380	1.11	0.87	0.44	0.47	364	42.34	39.64	0.66	0.94
		2430	1.04	1.14	0.51	0.5	363	48.08	49.04	0.69	1.02
**JF-1 (Jafr Basin)**	Mudawwara	1056	0.94	0.4	3	0.2	428	22.3	314.9	0.1	14.1
		1059	1.06	0.55	2.9	0.3	426	29.2	276.4	0.2	9.5
		1060	1.14	0.6	3.3	0.2	424	20.2	291.2	0.2	14.4
		1061	1.48	0.71	5	0.3	425	21.6	339.2	0.1	15.7
		1062	2.15	1.8	9.6	0.4	426	18.6	448.8	0.2	24.1
		1064	2.96	2.1	13.5	0.5	425	17.9	454.4	0.1	25.4
		1065	5.84	1.9	32.5	0.8	417	13.7	556.3	0.1	40.6
		1066	5.91	2.4	30.5	0.8	414	13.4	515.4	0.1	38.6
		1067	9.6	6.3	55.3	0.8	417	8	576.3	0.1	71.9
		1070	6.2	3.2	17.2	0.6	428	10	277.3	0.2	27.7
**HZ-4 (Azraq Basin)**	Ghareb	956.3	9.41	6.46	63.47	0.79	430	8.4	674.5	0.1	80.3
		957.9	9.26	6.65	60.34	0.69	429	7.5	651.6	0.1	87.4
		958.8	10	7.6	59.92	1.09	424	10.9	599.2	0.1	55
		959.8	12.8	10.33	76.2	1.11	427	8.7	595.3	0.1	68.6
		960.3	11.2	9.05	96.55	1.15	429	10.3	862.1	0.1	84
		961.2	11.47	9.61	70.06	0.01	428	0.1	610.8	0.1	7006
		963.5	10.57	9.26	68.76	0.88	427	8.3	650.5	0.1	78.1
		963.8	7.34	6.89	51.77	0.86	428	11.7	705.3	0.1	60.2
		964.2	14.06	9.22	76.4	1.09	424	7.8	543.4	0.1	70.1
	Wadi Essir	3297.5	2.52	1.6	8.42	0.4	448	15.9	334.1	0.2	21.1
		3298.8	1.82	1.11	4.04	0.23	449	12.6	222	0.2	17.6
**WS-3 (Sirhan Basin)**	Mudawwara	1389	1	0.19	0.94	0.43	438	43	94	0.17	2.19
		1482	1.35	0.24	1.24	0.4	441	29.63	91.85	0.16	3.1
		1484	1.33	0.24	1.17	0.4	439	30.08	87.97	0.17	2.93
		1489	3.12	0.44	3.78	0.44	437	14.1	121.15	0.1	8.59
		1494	3.33	0.48	3.89	0.48	438	14.41	116.82	0.11	8.1
	Dubeidib	1500	3.42	0.72	5.04	0.43	435	12.57	147.37	0.13	11.72
		1505	1.17	0.56	2.02	0.4	440	34.19	172.65	0.22	5.05
	Hiswa	2080	1.01	0.68	1.1	0.39	444	38.61	108.91	0.38	2.82
		2085	1.02	0.73	1.24	0.37	445	36.27	121.57	0.37	3.35
		2095	1.04	0.7	1.01	0.4	443	38.46	97.12	0.41	2.53

The TOC vs. S1 plot^37^ indicates that all the studied samples from different formations fall within the indigenous petroleum zone (Fig. 13b). A very few samples from the Ghareb and Mudawwara formations, in HZ-4 and RH-23, indicate that non-indigenous hydrocarbon may be present (Fig. 13b). The Tmax vs. PI plot has been used in the evaluation of the maturity of the studied samples^38^. The majority of the examined specimens from Ghareb (HZ-4), Mudawwara (JF-1 and WS-3), and Dubeidib (WS-3) lie in the mature oil zone (Fig. 13c). The Mudawwara sample from RH-23 of the Risha Basin was found to be immature (Fig. 13c). Wadi Essir samples of HZ-4 (Azraq Basin) and Hiswa samples of WS-3 (Sirhan Basin) indicate over-mature condensate zones (Fig. 13c).

 Shows the measured HI of all the specimens, providing insights into the generation potential of various hydrocarbon types. The Ghareb Formation in HZ-4 well has a HI ranging from 543.4 to 862.1 mg HC/g TOC, indicating an oil-prone type I/II kerogen (Fig. 13c). The Wadi Essir Formation in the same well has a HI range of 222 to 334.1 mg HC/g TOC, indicating an oil-prone type II/III kerogen capable of generating both oil and gas. The Mudawwara Formation in RH-23 well has most samples with a HI value ranging from 50 to 200 mg HC/g TOC, indicating type III kerogen, which is gas-prone. In the Mudawwara Formation of the JF-1 well, the samples have a HI value range of 277.3 to 556.3 mg HC/g TOC, indicating type II/III kerogen, which is capable of generating both oil and gas. On the other hand, the Mudawwara Formation in the WS-3 well has samples with a HI value range of 87.97 to 121.15 mg HC/g TOC, indicating a gas-prone type III kerogen.

By plotting the data on the HI-Tmax diagram, the Kerogen type and maturity can be assessed^39^. The Ghareb Formation in the HZ-4 well lies within the mature condensate wet gas window. The Wadi Essir Formation lies in the overmature dry gas window in the Tmax-HI plot (Fig. 13d). Most of the Mudawwara samples in RH-23 fall within the immature Type-III/IV kerogen zone. In contrast, part of these samples lies in the condensate/ wet gas window in the Tmax-HI plot (Fig. 13d). The Mudawwara Formation in both JF-1 and WS-3 wells, Dubeidib and Hiswa formations in WS-3 well are in the condensate wet gas window (Fig. 13d). Fig. 13e presents the HI vs OI cross plot (modified van Krevelen diagram). The Ghareb and Wadi Essir samples in HZ-4 exhibit type-I/II kerogens, while most samples of Mudawwara Formation in RH-23 belong to Type-III kerogen, and partly the mixed type III/II (Fig. 13e). The Mudawwara samples in JF-1 are of Type-I and Type-II kerogens, while Mudawwara, Dubeidib, and Hiswa formations in WS-3 well are of Type-II and Type-III kerogens (Fig. 13e). Based on the TOC-HI cross plot presented in Fig. 13d/f, the Ghareb and wadi Essir formations in HZ-4, and Mudawwara Formation in JF-1 are inferred as fair oil source rocks. The Mudawwara Formation in the Risha Basin (RH-23) and the Dubeidib and Hiswa formations in the Sirhan Basin (WS-3) are mixed gas and/or oil source to gas source (Fig. 13f).

### Burial history and thermal maturity

**Fig. 14:**
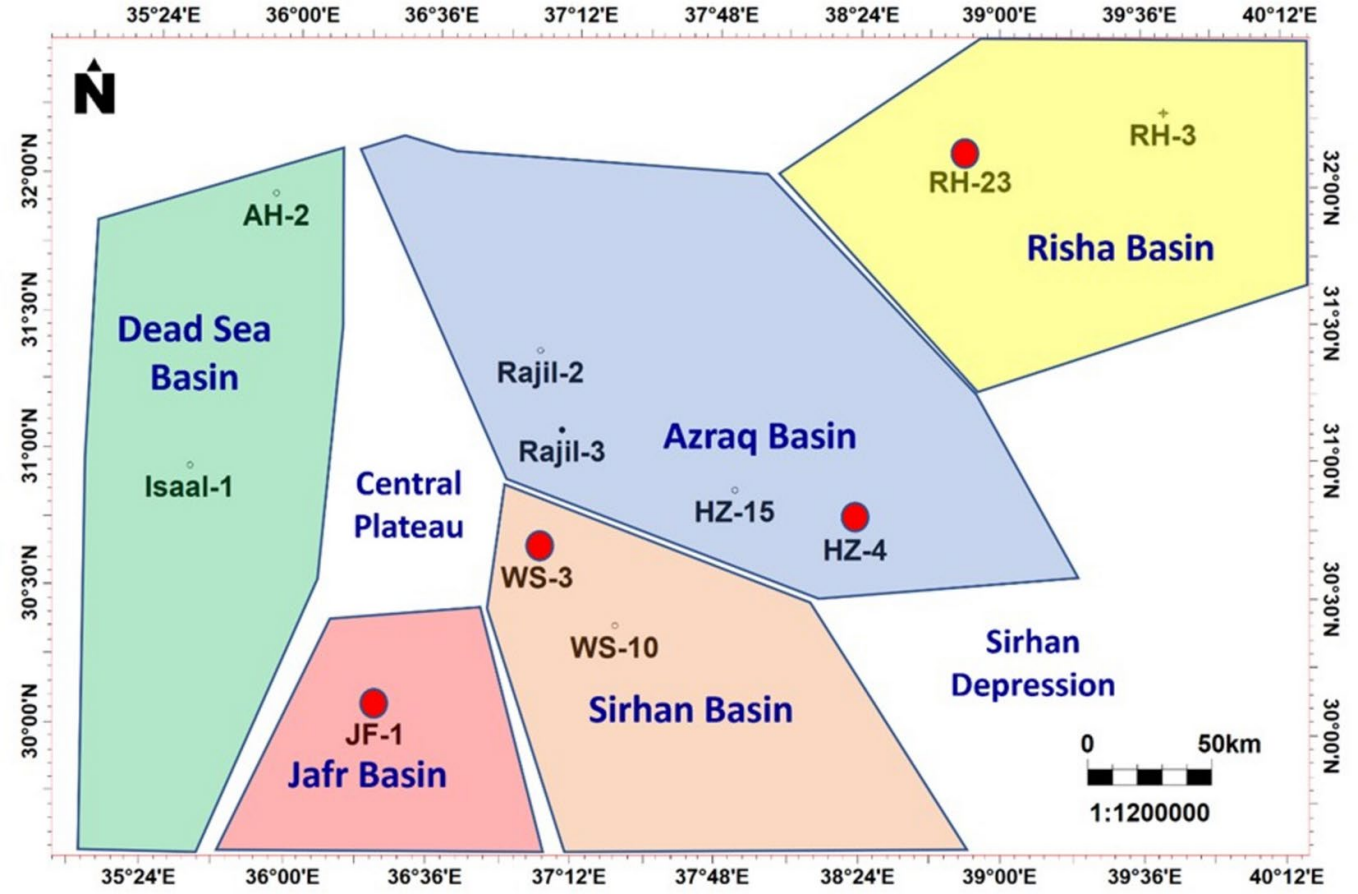
Location map showing the location of four wells (marked by red circles) with organic geochemical analysis, which were used for constructing the burial history models. These wells are HZ-4 from Azraq Basin, RH-23 from Risha Basin, JF-1 from Jafr Basin, and WS-3 from Sirhan Basin.

**Fig. 15:**
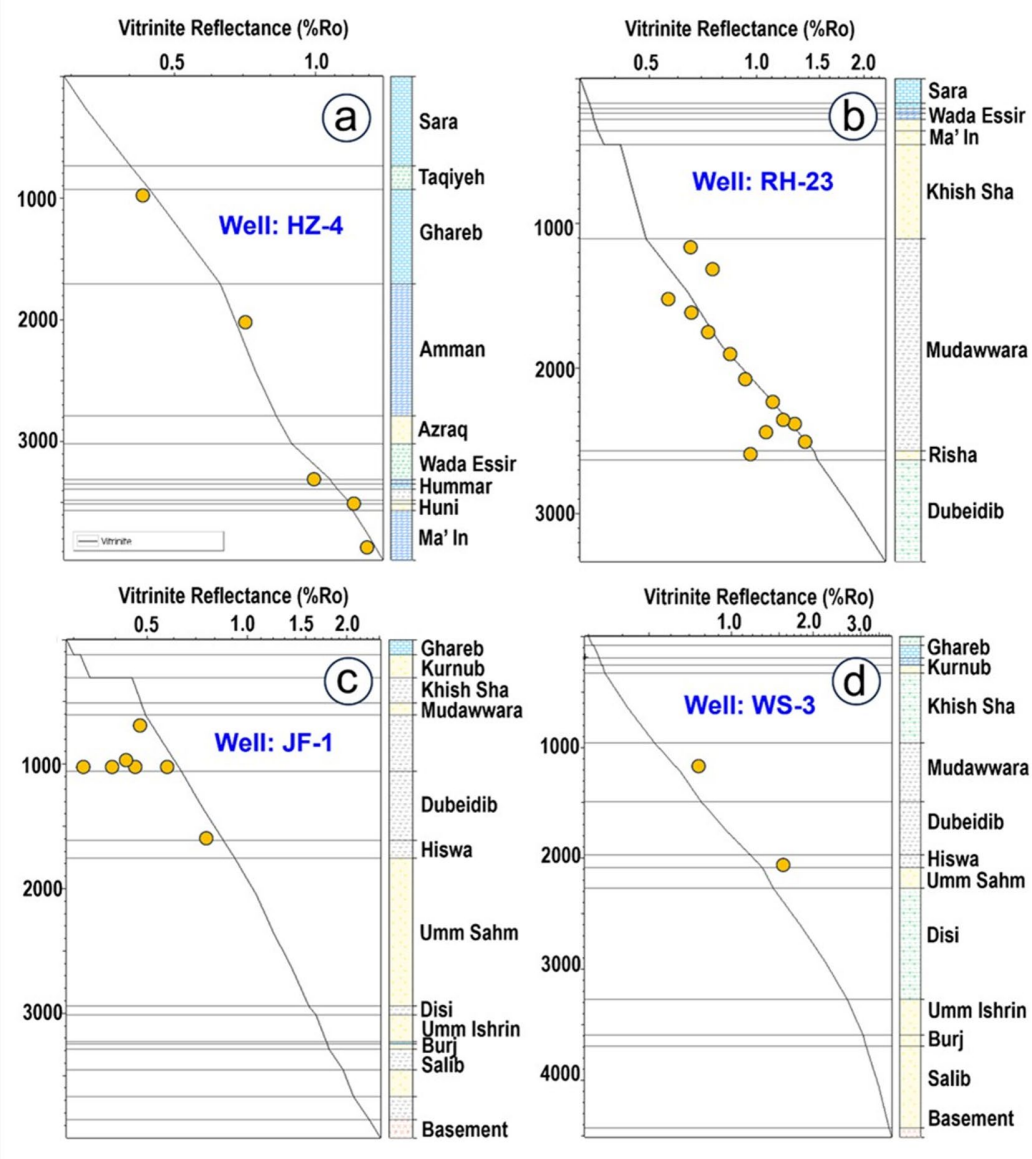
Calibrated 1D maturity models based on vitrinite reflectance (% Ro) data for (**a**) HZ-4 well from Azraq Basin, (**b**) RH-23 from Risha Basin, (**c**) JF-1 from Jafr Basin, and (d) WS-3 from Sirhan Basin. The X-axis plots vertical depth in meters.

Four wells were used in constructing the burial history models at different basins in Jordan (Fig. 14). These wells are HZ-4 in the Azraq Basin, RH-23 in the Risha Basin, WS-3 in the Sirhan Basin, and JF-1 in the Jafr Basin. The 1D maturity models, presented in Fig. 15, were constructed to assess the thermal maturity and expulsion of hydrocarbons for the studied source rocks, including Ghareb, Wadi Essir, Mudawwara, Dubeidib, and Hiswa formations. The geochemical data, including average TOC wt.%, average hydrogen index (HI), and geological data, including unit tops and bottoms, thicknesses, and unconformities, were integrated for the modelling purpose. These data are presented in Table 3. The measured average vitrinite reflectance values used in calibrating the modelled maturity profile are listed in Table 4.

**Table 3:** Formation tops, bases, and eroded sections (in meters as measured depth), along with corresponding ages of deposition and erosion (in million years, Ma), and formation lithologies for the RH-23, JF-1, HZ-4 and WS-3.

**Well RH-23**								
**Formation**	**Top**	**Base**	**Eroded**	**Deposition (Ma)**		**Eroded (Ma)**		**Lithology**
	**(m)**	**(m)**	**(m)**	**from**	**to**	**from**	**to**	
SARA FM	0	171	500	56	20	20	0	Limestone (ooid grainstone)
TAQIYEH FM	171	211		66	56			Limestone (micrite)
GHAREB FM	211	239		72	66			Dolomite (typical)
WADI ESSIR FM	239	285		100.5	72			Dolomite (organic lean, sandy)
KURNUB FM	285	362		152	100.5			Sandstone (typical)
MA’IN FM	362	455		251.9	251.2			Sandstone (clay rich)
KISH SHA	455	1109	1500	433.2	285	285	260	Sandstone (clay poor)
MUDAWWARA FM	1109	2577		458.4	433.2			Shale (organic rich, typical)
RISHA FM	2577	2636		461	458.4			Sandstone (clay poor)
DUBEIDIB FM	2636	3339		485.4	461			Siltstone (organic lean)
**Well JF-1**								
GHAREB	0	124		83.6	72.1			Limestone (micrite)
KURNUB FM	124	306	250	113	100.5	100.5	83.6	Sandstone (typical)
KHISH SHA FM	306	514	1000	427	419	419	113	Shale (organic lean, sandy)
MUDAWWARA SST	514	610		433.4	427			Sandstone (quartzite, typical)
MUDAWWARA CLAY	610	1073		445.2	433.4			Shale (organic lean, typical)
DUBEIDIB FM	1073	1642		470.7	445.2			Shale (organic rich, typical)
HISWA FM	1642	1785		485	470.7			Shale (organic lean, typical)
UMM SAHM FM	1785	2987		494	489.5			Sandstone (typical)
DISI FM	2987	3059		497	494			Shale (typical)
UMM ISHRIN FM	3059	3275		500.5	497			Sandstone (clay poor)
BURJ FM	3275	3292		504.5	500.5			Limestone (micrite)
BURJ SST	3292	3336		509	504.5			Sandstone (clay poor)
SALIB FM	3336	3500		514	509			Shale (organic lean, typical)
SALIB SST	3500	3718		521	514			Sandstone (clay poor)
SALIB CLAY	3718	3900		541	521			Shale (organic rich, typical)
BASALT	3900	4048		560	541			Basalt (normal)
**Well HZ-4**								
SARA FM	0	742	200	56	33	33	0	Limestone (shaly)
TAQIYEH FM	742	933		66	56			Marl
GHAREB FM	933	1712	50	70	67	67	66	Limestone (micrite)
AMMAN FM	1712	2798		83	70			Dolomite (typical)
AZRAQ FM	2798	3028		86.3	83			Sandstone (typical)
WADI ESSIR FM	3028	3323		93.9	86.3			Marl
SHUEIB FM	3323	3363		95	93.9			Dolomite (typical)
HUMMAR FM	3363	3400		98	95			Limestone (ooid grainstone)
FUHAYS FM	3400	3491		113	98			Shale (typical)
KURNUB FM	3491	3523	200	163.5	115	115	113	Sandstone (typical)
HUNI FM	3523	3576		174.1	163.5			Sandstone (clay poor)
MAIN FM	3576	3984		251.9	201.3			Dolomite (typical)
**Well WS-3**								
TAQIYEH FM	0	84	200	60	55	55	0	Marl
GHAREB FM	84	198	500	75	70	70	60	Limestone (ooid grainstone)
AMMAN FM	198	260		83.6	75			Dolomite (typical)
KURNUB FM	260	335	400	113	100.5	100.5	83.6	Sandstone (typical)
KHISH SHA FM	335	964		427	419			Siltstone (organic lean)
MUDAWWARA FM	964	1499		443.8	427			Shale (organic rich, typical)
DUBEIDIB FM	1499	1985		458.4	443.8			Shale (organic rich, typical)
HISWA FM	1985	2100		470	458.4			Shale (organic rich, typical)
UMM SAHM	2100	2285		477.7	470			Sandstone (typical)
DISI FM	2285	3290		485.4	477.8			Siltstone (organic lean)
UMM ISHRIN FM	3290	3609		509	485.4			Sandstone (clay rich)
BURJ FM	3609	3710		521	509			Sandstone (clay poor)
SALIB FM	3710	4450		541	521			Sandstone (typical)
BASEMENT	4450	4530		570	541			Basalt (normal)

**Table 4:** Measured vitrinite reflectance (Ro) values at various depths in RH-23, JF-1, HZ-4 and WS-3 wells. Depths are measured depths (MD).

**Well**	**Depth (m)**	**Ro %**
**RH-23**	1160	0.65
	1530	0.56
	1600	0.66
	1740	0.72
	2080	0.93
	2250	1.12
	2360	1.17
	2430	1.04
	2593	0.94
**HZ-4**	964.2	0.43
	2010	0.7
	3297.5	1
	3525	1.2
	3871	1.29
**WS-3**	189	0.29
	1183	0.73
	2061	1.5
**JF-1**	711	0.47
	1043	0.45
	1059	0.38
	1032	0.42
	1066	0.31
	1076	0.58
	1630	0.74

The four burial history models are well calibrated as shown in Fig. 15, where the maximum modelled maturity for HZ-4 well, in Azraq Basin, equals 1.4 Ro% at Ma’In Formation (Fig. 15a). The maximum modelled maturity for RH-23 in Risha Basin equals 2.2 Ro% at Dubeidib Formation (Fig. 15b). In comparison, the Salib Formation in JF-1 well of Jafr Basin shows 2.5 Ro% (Fig. 15c). The maximum modelled maturity for WS-3 well, in Sirhan Basin, equals 3.5 Ro% at Salib Formation (Fig. 15d). In Azraq Basin, two potential source rocks were evaluated in HZ-4 well within Ghareb limestone and Wadi Essir shales (Table. 2, Fig. 13).

**Fig. 16:**
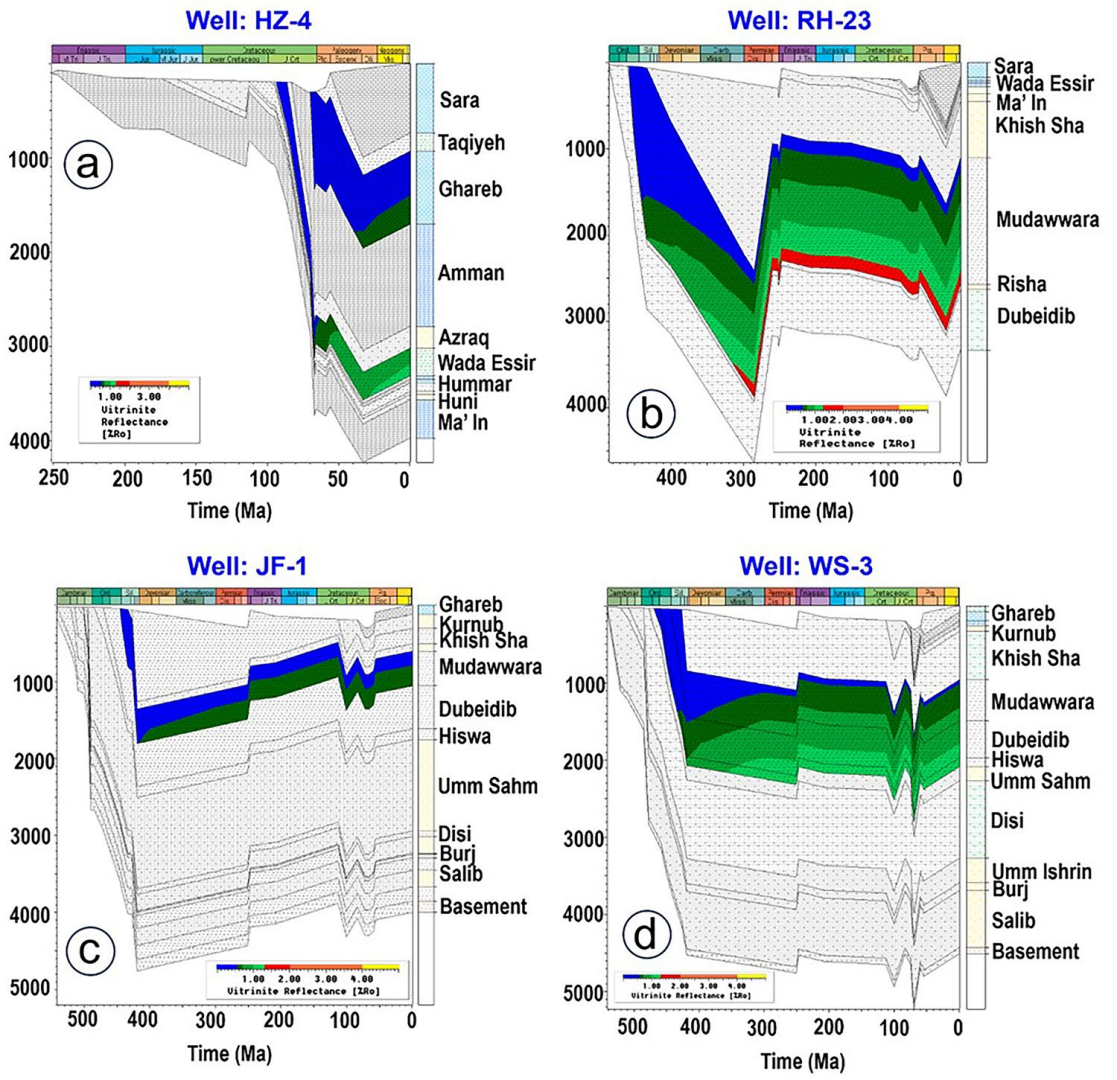
Burial history model construction for (**a**) HZ-4 well from Azraq Basin, (**b**) RH-23 from Risha Basin, (**c**) JF-1 from Jafr Basin, and (**d**) WS-3 from Sirhan Basin.

The burial history model of HZ-4 well showed that most of the Ghareb Formation is immature. At the same time, the lowermost part entered the early oil window (0.55-0.7 Ro%) at about 50 Ma ago (~ 1700 m depth), and then no hydrocarbons were expelled (Fig. 16a). On the other hand, most of the Wadi Essir Formation is mature in the same well, where it entered the main oil window (0.7-1 Ro%) at about 60 Ma ago (~ 3000 m depth) and entered the late oil window (0.7-1 Ro%) at about 32 Ma ago (~ 3500 m depth) (Fig. 16a). The burial history model of RH-23 well showed that most of Mudawwara Formation is mature, where it was entered the main oil window (0.7-1 Ro%) at about 420 Ma ago (~ 2000 m depth), and then in the late oil window (1-1.3 Ro%) at about 370 Ma ago (~ 2800 m depth), and finally the wet gas window (1-1.3 Ro%) at about 320 Ma ago (~ 3400 m depth) (Fig. 16b).

The burial history model of JF-1 well that most of the Mudawwara Formation is immature. The lowermost part entered the early oil window (0.55-0.7 Ro%) at about 420 Ma ago (~ 1800 m depth), and then no hydrocarbons were expelled (Fig. 16c). The burial history model of WS-3 well showed that the lowermost part of Mudawwara Formation is mature, where it entered the main oil window (0.7-1 Ro%) at about 340 Ma ago (~ 1500 m depth) as shown in Fig. 16d. The Dubeidib Formation is mature in the same well, where was entered the main oil window (0.7-1 Ro%) at about 420 Ma ago (~ 2000 m depth) and entered the late oil window (1-1.3 Ro%) at about 340 Ma ago (~ 2100 m depth). On the other hand, the Hiswa source rocks entered the main oil window (0.7-1 Ro%) at about 430 Ma ago (~ 2100 m depth) and entered the late oil window (1-1.3 Ro%) at about 350 Ma ago (~ 2200 m depth), as shown in Fig. 16d.

**Fig. 17:**
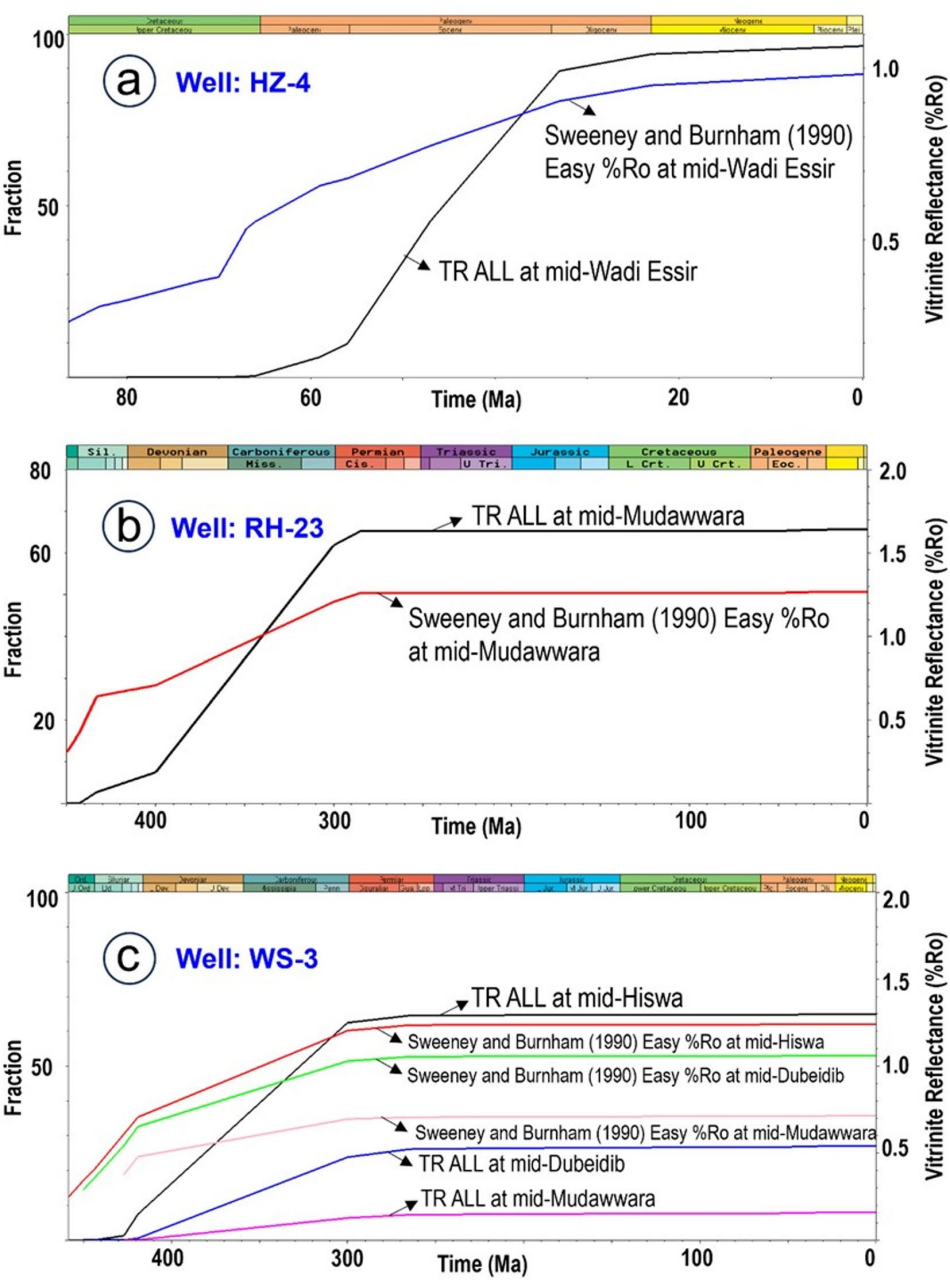
The modeled transformation ratio (TR) and vitrinite reflectance (%Ro) with time for (**a**) HZ-4 well from Azraq Basin, (**b**) RH-23 from Risha Basin, and (**c**) WS-3 from Sirhan Basin.

The modeled transformation ratio (TR) through time for the mature source rocks in the HZ-4, RH-23, and WS-3 wells is shown in Fig. 17. The critical moment of petroleum generation for the Wadi Essir source rock in the Azraq Basin, HZ-4 well, occurred in the Late Eocene (48 Ma ago) as shown in (Fig. 17a). The critical moment of petroleum generation for the Mudawwara source rock in the Risha Basin, RH-23 well, occurred in the Middle Carboniferous (350 Ma ago) as shown in (Fig. 17b). The critical moment of petroleum generation for the Hiswa source rock in the Sirhan Basin, WS-3 well, occurred in the Late Carboniferous (340 Ma ago), while the transformation ratio of both Mudawwara and Dubeidib source rocks in the Sirhan Basin is below 50 % (Fig. 17c).

## Discussion

Reservoir quality across the Azraq, Dead Sea, Sirhan, and Risha basins is highly heterogeneous, reflecting the combined influence of depositional architecture, diagenetic overprint, and structural framework. Heterogeneity at the basin scale is principally governed by lateral and vertical variations in facies distribution, subsequent diagenetic modification (cementation, dissolution, compaction, and authigenic mineral growth), and the presence or absence of structurally defined compartments. Where depositional systems produce thick, laterally continuous sand bodies and diagenesis preserves primary intergranular porosity or generates secondary porosity, reservoirs tend to exhibit higher effective porosity and hydrocarbon saturation. Conversely, facies pinch-outs, pervasive silica cementation, and pressure-solution features commonly reduce porosity and permeability, producing compartmentalized and low-permeability intervals that require targeted characterization and stimulation to be productive. Integrating regional geological context with well-scale petrophysical data yields a more refined assessment of reservoir effectiveness, clarifying the principal controls on storage and flow.

### Basin-wise reservoir petrophysical characteristics and implications

- The Risha Basin emerges as the most prospective of the studied basins. Petrophysics confirms the presence of multiple stacked pay zones in Dubeidib, Risha, and Umm Sahm formations. These units, particularly the Umm Sahm Formation, contain up to 100 meters of thick sandstone pay intervals, comprising laterally extensive clean sandstone bodies and sand-rich intervals, implying deposition in fluvial to shallow marine settings. Petrographically and diagenetically, the Palaeozoic reservoirs consist of quartz arenites and subarkoses with high mineralogical maturity (75-95% quartz), indicating a continental crustal origin, similar to that of the Arabian Shield^40,41^. Reservoir quality is strongly controlled by silica cementation, pressure solution (i.e., stylolites), and extensive quartz overgrowths^42^. These processes significantly reduce original porosity and limit the development of secondary porosity, particularly in the Umm Sahm Formation. The studied formations exhibit very high hydrocarbon saturation, ranging from 70% to 80%. Although matrix properties are commonly tight, with matrix porosity typically ~7-12% and matrix permeability often <1 mD, natural fracture networks and localized permeability enhancement are decisive for commercial flow. Image logs from wells RH-7 and RH-16 reveal open or partially open fractures that correlate with improved productivity, underscoring the importance of fracture characterization in development planning^7^. The structural setting, including fault-bounded blocks and paleohighs, further enhances trap integrity and charge preservation. Collectively, these attributes are consistent with the interpretation of the Risha accumulations as tight-gas systems that can yield economic volumes where fracture connectivity, structural closure, and appropriate reservoir development strategies are present^4,40^. The Risha case illustrates that low matrix porosity and permeability do not preclude commercial production when favourable structural and diagenetic conditions coincide with effective fracture networks and laterally continuous reservoir facies. For exploration and field development, this implies prioritizing: (1) high-resolution structural mapping to identify structural sweet spots; (2) detailed fracture and stress-field characterization to target naturally permeable and rock-mechanically suitable corridors; and (3) reservoir-specific stimulation designs (including horizontal well placement) to connect and exploit stacked pay intervals. There are two types of traps: 1) Conventional play within Risha Field, consisting of shallow marine to fluviatile sediments within a channel complex and trapped within a larger four-way dip structure. The extent of the reservoir sweet spot could account for the current production and well results. 2) Glacial play on the flank of the Risha structure outside the regional structural closure. Seismic data quality precludes confirmation of the presence of such glacial ‘tunnel’ valleys in the area. This play is one of the key objectives of the study that screens for prospectivity outside the Risha field. A key play concept identified is related to Late Ordovician to Early Silurian glaciations. The glacial play, comprising peri-glacial systems ranging from incised valleys along the shelf in North Saudi Arabia and Jordan to marine and unconfined submarine fans further outboard to the North.
- Discrete pay intervals within the Azraq Basin, notably in the Shueib and Wadi Essir formations, exhibit limited reservoir potential at the field scale. These Late Cretaceous shallow marine, intrashelf-restricted, and intrashelf-basin carbonates are characterized by elevated total organic carbon and locally self-sourcing behaviour^5,16,43^. Recent petrophysical logs from wells HZ‑15 and Rajil‑2 record porosities of 8–15%, shale volumes of < 5%, and hydrocarbon saturations of 50-60%, indicating that individual intervals can retain hydrocarbons and may form viable traps. However, net pay is typically thin (10-12 m), and lateral continuity is limited, likely reflecting local facies variability, early diagenetic cementation, or later diagenetic sealing. Dolomitization and fracture networks locally enhance permeability. However, the reservoir architecture is more compartmentalized and discontinuous than in the Risha Basin, favoring selective, prospect-scale targets rather than broad-field development.
- Reservoir properties in the Dead Sea Basin are highly variable, particularly within the Salib and Umm Rijam formations. The Salib Formation is typically sourced from the overlying or downdip lacustrine shales or from the Middle Cambrian Burj Formation, where organic-rich shale facies are present. Certain intervals display favorable metrics—porosities of 10–17% and hydrocarbon saturations of up to ~80%, whereas adjacent intervals approach economic cutoffs (porosity of ~7%; shale of up to ~30%). This intraformational variability is attributable to complex depositional dynamics (fluctuating marine energy and facies transitions) and to diagenetic processes such as cementation and mechanical compaction at greater burial depths. Although the Umm Rijam is commonly chalky and tight, it may function as an unconventional reservoir where fracture density is high and sealing facies preserve charge. The Umm Rijam interval is frequently associated with the Muwaqqar Chalk Marl Formation, where highly superior oil shale deposits have been discovered. As a tight unconventional reservoir in buried environments, the Umm Rijam Formation can be exploited, subject to proper maturation and fracture-enhancement techniques.
- The Salib Formation, as encountered in well WS‑10 (Sirhan Basin), exhibits moderate reservoir quality characterized by an average porosity of ~10%, shale volume near 15%, and roughly balanced hydrocarbon/water saturations. The principal reservoir interval is relatively thin (~10 m). Although the sandstones retain acceptable petrophysical attributes, their volumetric significance is constrained by the absence of multiple stacked horizons, unlike the Risha succession, thereby limiting large-scale, cumulative field potential. Petrographic investigation of Salib sandstones from WS‑3 indicates a detrital assemblage dominated by moderately to well‑rounded feldspars and well‑rounded to subangular quartz with moderate to high sphericity, consistent with a fluvial to shallow‑marine depositional regime. Porosity is predominantly primary intergranular, with a subordinate component of secondary porosity generated by dissolution of feldspar and, locally, quartz. Diagenetic modification, primarily through quartz overgrowths and clay cements, has reduced the effective pore volume and occluded pore throats, thereby diminishing permeability. From an exploration and development perspective, the Salib interval should be regarded as a prospect‑scale target: reservoir success will depend on identifying loci where structural closure or favorable diagenetic alteration (e.g., enhanced dissolution, localized dolomitization, or fracture networks) preserves or augments permeability. Detailed core-scale and thin-section analyses, combined with targeted petrophysical and fracture characterization, are recommended to delineate sweet spots and inform well placement and completion strategies.

### Source rock perspective and regional variations

Rock‑Eval pyrolysis indicates a suite of indigenous source intervals across the study area. In the Azraq Basin, the Ghareb and Wadi Essir formations exhibit geochemical signatures consistent with oil‑prone source rocks. The Mudawwara Formation is the principal organic‑rich unit in the Risha, Jafr, and Sirhan basins. In contrast, the Dubeidib and Hiswa formations represent the main source intervals within the Sirhan Basin. These distributions reflect basin-scale depositional and preservation patterns that control the accumulation of organic matter and early diagenetic alteration. All these source rocks are dominantly indigenous in origin. Kerogen typing from Rock-Eval data indicates that the Ghareb and Wadi Essir shales are dominated by Type I/II kerogen, suggesting a strong propensity for oil generation under suitable thermal conditions. The Dubeidib and Hiswa formations in the Sirhan Basin are primarily fair to good oil and gas source rocks.

Modelled thermal maturity, calibrated against measured vitrinite reflectance, correlates closely with basin burial depth. The Sirhan Basin attains the highest modelled maturity (up to ~3.5 %Ro), consistent with a greater depth to basement (~4,500 m). By contrast, the Jafr Basin reaches modelled maturities of ~2.5% Ro at a basement depth of ~3,900 m, despite having a similar geologic age in both wells (541 Ma). The Azraq and Risha basins record lower modeled maturities (approximately 1.4% Ro and 2.2% Ro, respectively); the basement was not encountered in the studied wells (HZ-4, RH-23), which constrains direct depth calibration in those sections. These maturity gradients control the timing and extent of hydrocarbon generation, expulsion, and secondary cracking.

Burial‑history reconstructions indicate that regional tectonothermal episodes have exerted a first‑order influence on maturation pathways. In the RH‑23 burial model, for example, the Hercynian orogeny (circa 320-280 Ma) is inferred to have accelerated thermal maturation. It may have promoted secondary cracking of oil to gas in deeply buried intervals^36^. In the Azraq Basin, Wadi Essir shales entered the principal oil window during the Eocene, whereas Mudawwara shales in the Risha Basin reached the main oil window in the Silurian. Mudawwara shales in the Jafr Basin entered early oil generation in the Devonian, but, according to the modeling, did not expel significant hydrocarbons, an outcome consistent with the fact that JF-1 was a dry hole. While no commercial hydrocarbons have been seen in initial drilling programs, the structural geometry of the Jafr Rift and the ideal sandstone facies continue to render the Salib Formation a frontier exploration target. Although several units (Mudawwara, Dubeidib, Hiswa) achieved main and late generation windows during the Late Paleozoic, their effectiveness as charge contributors differs. The Hiswa Formation, with a modeled transformation ratio exceeding 50% since the Middle Carboniferous, is identified as the most effective generator in the Sirhan Basin. The transformation ratio, together with the timing of maturation relative to trap formation and migration pathways, therefore, provides a critical discriminator of which source intervals are likely to have contributed to the commercial charge.

The integrated geochemical and basin‑modeling results emphasize that successful charge prediction requires coupling kerogen quality with robust maturity and burial histories. Exploration strategies should prioritize: (1) mapping of mature source kitchens (high transformation ratios and appropriate timing), (2) assessment of migration pathways and seal integrity, and (3) calibration of basin models with measured vitrinite reflectance and well data to reduce uncertainty in phase prediction. These steps will improve the targeting of prospects most likely to have received and retained economically significant hydrocarbon accumulations.

## Conclusions

This study assesses the reservoir characteristics, source rock properties, and infers burial history and calibrated maturity models across the Azraq, Dead Sea, Sirhan, Jafr, and Risha basins by integrating available petrographic data, wireline logs, and organic geochemistry data.

- The Risha Field has been identified as the most favorable area, which hosts many thick, amalgamated, clean, and hydrocarbon-saturated intervals in the Risha, Dubeidib, and Umm Sahm formations, indicating favorable exploration and development potential, subject to permeability, fracture connectivity, and production-test confirmation.
- Azraq Basin exhibits localized potential in the Shueib and Wadi Essir intervals. Limited lateral continuity, dolomitization, and fracturing create isolated sweet spots.
- The Dead Sea Basin exhibits strong intraformational variability; some intervals display good porosity (10–15%) and saturation, while adjacent zones approach economic cutoffs. Fracture density and diagenetic sealing govern unconventional potential.
- Sirhan Basin hosts moderate quality reservoirs with thin pay and limited stacking; petrography indicates fluvial–shallow marine provenance with diagenetic reduction of primary porosity.
- Ghareb and Wadi Essir (Azraq) are oil-prone; Mudawwara, Dubeidib, and Hiswa show mixed oil–gas potential. Modelled maturity correlates with basin depth and controls charge timing and phase.

The integrated petrophysical, petrographic, and basin-modeling framework developed here refines the understanding of reservoir distribution and quality across the sudies basins, providing a practical foundation for prospect selection, field development optimization, and risk-informed drilling decisions. The observed spatial variations in reservoir properties reflect the need for continued high‑resolution geological, geophysical, and geochemical work to convert identified potential into reliably producible volumes. The conclusions of this study provide a useful premise for subsequent exploration activities, field development optimization, and zone selection for drilling and reservoir management in the studied basins.

## Data Availability

The data utilized in this study are confidential and cannot be shared publicly. Please get in touch with the corresponding author if you have any questions.
